# Evaluation of harvest and information needs for North American sea ducks

**DOI:** 10.1371/journal.pone.0175411

**Published:** 2017-04-18

**Authors:** Mark D. Koneff, Guthrie S. Zimmerman, Chris P. Dwyer, Kathleen K. Fleming, Paul I. Padding, Patrick K. Devers, Fred A. Johnson, Michael C. Runge, Anthony J. Roberts

**Affiliations:** 1 Division of Migratory Bird Management, U.S. Fish and Wildlife Service, Orono, Maine, United States of America; 2 Division of Migratory Bird Management, U.S. Fish and Wildlife Service, Sacramento, California, United States of America; 3 Division of Migratory Birds, U.S. Fish and Wildlife Service, Hadley, Massachusetts, United States of America; 4 Division of Migratory Bird Management, U.S. Fish and Wildlife Service, Laurel, Maryland, United States of America; 5 Wetland and Aquatic Research Center, U. S. Geological Survey, Gainesville, Florida, United States of America; 6 Patuxent Wildlife Research Center, U. S. Geological Survey, Laurel, Maryland, United States of America; Université de Sherbrooke, CANADA

## Abstract

Wildlife managers routinely seek to establish sustainable limits of sport harvest or other regulated forms of take while confronted with considerable uncertainty. A growing body of ecological research focuses on methods to describe and account for uncertainty in management decision-making and to prioritize research and monitoring investments to reduce the most influential uncertainties. We used simulation methods incorporating measures of demographic uncertainty to evaluate risk of overharvest and prioritize information needs for North American sea ducks (Tribe *Mergini*). Sea ducks are popular game birds in North America, yet they are poorly monitored and their population dynamics are poorly understood relative to other North American waterfowl. There have been few attempts to assess the sustainability of harvest of North American sea ducks, and no formal harvest strategy exists in the U.S. or Canada to guide management. The popularity of sea duck hunting, extended hunting opportunity for some populations (i.e., special seasons and/or bag limits), and population declines have led to concern about potential overharvest. We used Monte Carlo simulation to contrast estimates of allowable harvest and observed harvest and assess risk of overharvest for 7 populations of North American sea ducks: the American subspecies of common eider (*Somateria mollissima dresseri*), eastern and western populations of black scoter (*Melanitta americana*) and surf scoter (*M*. *perspicillata*), and continental populations of white-winged scoter (*M*. *fusca*) and long-tailed duck (*Clangula hyemalis*). We combined information from empirical studies and the opinions of experts through formal elicitation to create probability distributions reflecting uncertainty in the individual demographic parameters used in this assessment. Estimates of maximum growth (*r*_max_), and therefore of allowable harvest, were highly uncertain for all populations. Long-tailed duck and American common eider appeared to be at high risk of overharvest (i.e., observed harvest < allowable harvest in 5–7% and 19–26% of simulations, respectively depending on the functional form of density dependence), whereas the other populations appeared to be at moderate risk to low risk (observed harvest < allowable harvest in 22–68% of simulations, again conditional on the form of density dependence). We also evaluated the sensitivity of the difference between allowable and observed harvest estimates to uncertainty in individual demographic parameters to prioritize information needs. We found that uncertainty in overall fecundity had more influence on comparisons of allowable and observed harvest than adult survival or observed harvest for all species except long-tailed duck. Although adult survival was characterized by less uncertainty than individual components of fecundity, it was identified as a high priority information need given the sensitivity of growth rate and allowable harvest to this parameter. Uncertainty about population size was influential in the comparison of observed and allowable harvest for 5 of the 6 populations where it factored into the assessment. While this assessment highlights a high degree of uncertainty in allowable harvest, it provides a framework for integration of improved data from future research and monitoring. It could also serve as the basis for harvest strategy development as management objectives and regulatory alternatives are specified by the management community.

## Introduction

Wildlife managers routinely seek to establish sustainable limits of sport harvest or other regulated forms of take while confronted with considerable uncertainty. A growing body of ecological research focuses on methods to describe and account for uncertainty in management decision-making and to prioritize research and monitoring investments to reduce the most influential uncertainties (for examples see[1–10]). We used simulation methods incorporating measures of demographic uncertainty to evaluate risk of overharvest and prioritize information needs for North American sea ducks (Tribe *Mergini*).

**Fig 1:**
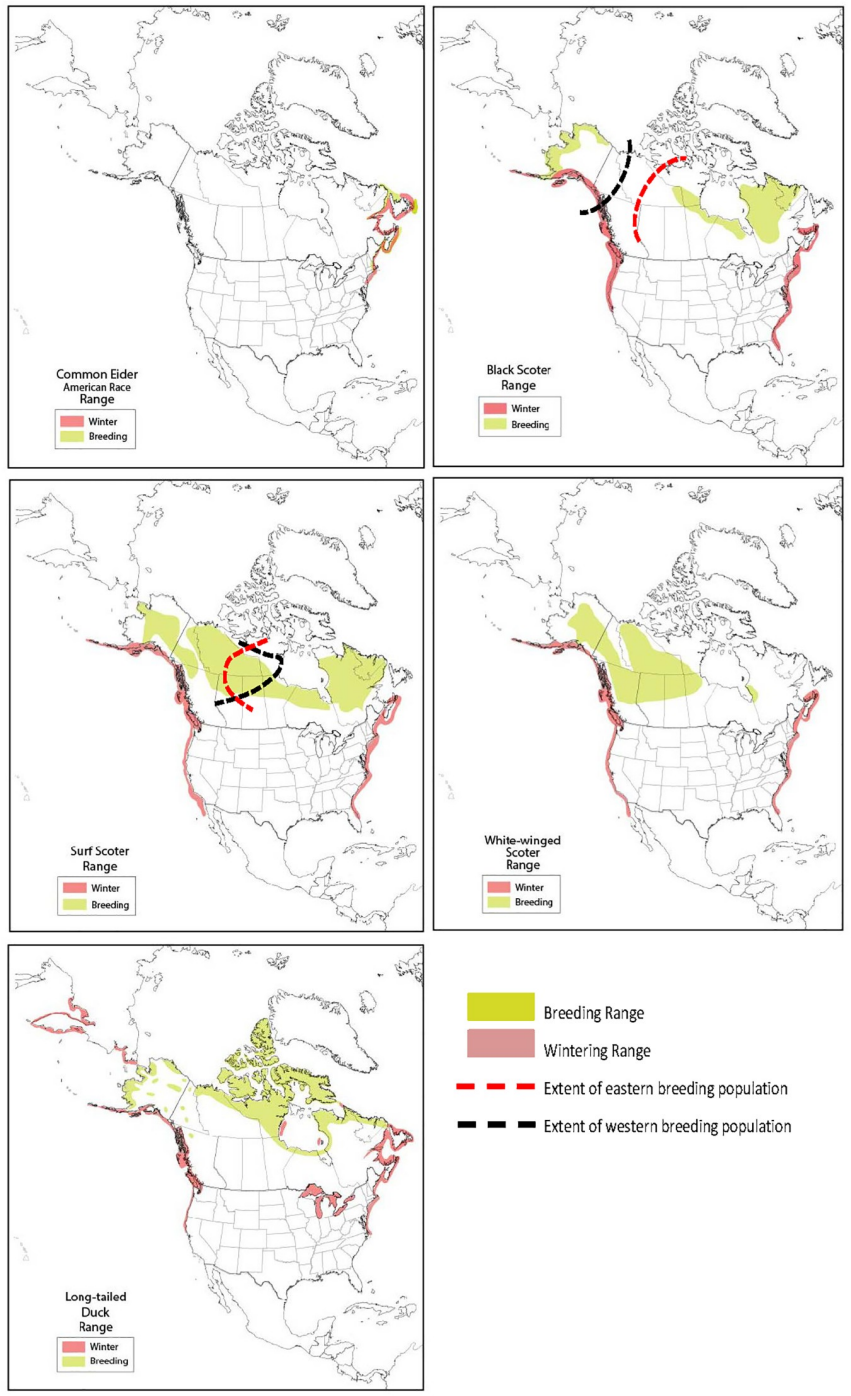
North American breeding and winter ranges of American common eider (*Sometaria mollissima dresseri*), eastern and western populations of black scoter (*Melanitta americana*) and surf scoter (*M*. *perspicillata*), white-winged scoter (*M*. *fusca*), and long-tailed duck (*Clangula hyemalis*).

There are 15 sea duck species endemic to North America and 22 populations recognized as allopatric or independent for purposes of management (Fig 1; [11]). Sea ducks are popular game birds in North America, yet they are poorly monitored and their population dynamics are poorly understood relative to other North American waterfowl [12–17]. Sea duck life histories are characterized by high adult survival, delayed maturation, and low reproductive capacity suggesting that population abundance of these species may be sensitive to factors influencing adult survival (e.g., harvest). Increased interest in sport harvest of sea ducks in some areas in recent decades may be related to regulatory restrictions on other species such as Canada goose (*Branta canadensis*) and American black duck (*Anas rubripes*). Special hunting regulations, established in the 1930s to increase hunting opportunity on sea ducks, reflect past perceptions that these species were lightly harvested and could sustain additional harvest pressure [12, 18]. However, limited population monitoring data for North American sea ducks suggest that 10 of the 15 species were declining in the 1980s and 1990s [12, 13–17]. More recent analyses indicated that 2 of the 22 recognized North American sea duck populations are currently declining, while the status of 9 populations is unknown [11]. The causes of past and present declines are largely unknown.

The popularity of sea duck hunting, extended hunting opportunity for some populations (i.e., special seasons and/or bag limits), and population declines have led to concern about potential overharvest. Owing to data limitations, there have been few attempts to assess the sustainability of harvest of sea ducks [19, 20]. We assessed risk of overharvest of sea ducks by contrasting estimates of allowable harvest to contemporary harvest estimates. Based on socio-economic importance of sport or subsistence harvest and concern over declining or unknown population status, we focused on 7 populations: the American subspecies of common eider (*Somateria mollissima dresseri*), eastern and western populations of black scoter (*Melanitta americana*) and surf scoter (*M*. *perspicillata*), and continental populations of white-winged scoter (*M*. *fusca*) and long-tailed duck (*Clangula hyemalis*). Satellite telemetry data suggests a degree of breeding range overlap for the surf scoter populations (Fig 1; Sea Duck Joint Venture, unpublished data) and limited exchange has been documented to occur between North American, European, and Asian populations for other species, however, we consider immigration/emigration negligible and do not address them here.

Quantification of uncertainty is crucial to evaluating the results of the assessment for harvest management as well as identifying and prioritizing information needs. We evaluate risk of overharvest for individual populations as an aid in evaluating contemporary harvest policies, conditional on the descriptions of uncertainty in demographic parameters presented. We also evaluated the influence of uncertainty in demographic parameters on conclusions about the risk of overharvest as a means to prioritize future research and monitoring to improve harvest decisions.

## Methods

Assessment of overharvest risk and prioritization of information needs for each population involved several steps. First, we combined published and unpublished information with expert opinion to define probability distributions for demographic parameters used in estimation of allowable harvest and contemporary levels of sport and subsistence harvest. Second, we used simulation to propagate uncertainty in individual demographic parameters into probability distributions for allowable harvest and for contemporary observed harvest. In the simulations, we compared estimates of allowable and observed harvest. Subsequently, we calculated the proportion of simulations where observed harvest < allowable harvest, as a measure of risk of overharvest. Finally, we used linear regression to assess the sensitivity of the simulated differences between allowable and observed harvests to uncertainty in individual demographic parameters in order to prioritize information needs.

### Prescribed take level framework

Limited biological and demographic information for the populations studied led us to select the Prescribed Take Level (PTL) [8] framework to assess allowable take. The PTL framework is based on the theory of density-regulated population growth [8, 21, 22]. Prescribed Take Level is a generalization of the Potential Biological Removal (PBR) framework [23] and is applicable to a broad range of take applications, including hunting. PBR was initially developed to regulate human-caused mortality of marine mammals [23] and both PBR and PTL methods have been applied to management of sport harvest as well as permitted and illegal take of birds [8, 22, 24–28].

Previous applications of PTL to birds were based on breeding population (i.e., pre-growth) estimates. For reasons detailed in *Developing Probability Distributions for Demographic Parameters*, we reformulated PTL for a post-growth (i.e., fall) population by deriving the maximum sustainable harvest rate for a fall population under the theta-logistic growth model (see S1 File for derivation). When annual estimates of post-growth population size are available, PTL can be applied to annual harvest management decisions as:

$${PTL}_{t}=H_{t}^{FF}=F_{0}\times \left(\frac{r_{max}\times \theta }{1+\theta (1+r_{max})}\right)\times N_{t}^{FF}$$ (1)

where PTL_*t*_ is the prescribed or allowable harvest level ($H_{t}^{FF}$, in numbers of individuals) for year *t*, $N_{t}^{FF}$ is fall population size in year *t*, *r*_max_ is the intrinsic rate of increase for the population, and *F*_o_ is a scaling factor representing the management objective, i.e., the desired take level relative to maximum sustained yield (MSY) [8, 22]. A harvest strategy with an objective of MSY would set *F*_0_ = 1, whereas *F*_0_ values <1 reflect a more conservative strategy, relative to risk of overharvest, with an associated equilibrium population size greater than that under a MSY strategy. The parameter *θ* allows for non-linear density dependence, which can influence allowable take [22, 29].

Alternatively, if harvest rate can be directly measured for a population, PTL for a post-growth population can be expressed as an allowable harvest rate by:

$$PTL=h_{MSY}^{FF}=F_{0}\times \left(\frac{r_{max}\times \theta }{1+\theta (1+r_{max})}\right)$$ (2)

A strategy based on harvest rate requires no associated measure of population size to ensure sustainability since harvesting at the allowable rate over time will cause population size to converge on the desired equilibrium state as dictated by *F*_0_. In contrast, PTL strategies based on total harvest must include a monitoring program capable of tracking changes in population size to appropriately scale harvest levels to population size. Strategies based on harvest rate are intrinsically robust to environmental stochasticity causing variation in carrying capacity. For some bird species, marking and recovery data obtained from banding or ringing programs can be used to estimate harvest and survival rates [30, 31]. Of the populations we evaluated, only common eider were sufficiently banded to permit a harvest-rate formulation of PTL. While the harvest rate-based PTL for eiders is specific to the female segment of the population, the total harvest-based PTL estimates for the other populations include both sexes.

No explicit harvest management objectives have been established for sea ducks other than the objective of sustainability implicit in the provisions of the Migratory Bird Treaty Act (16 U.S.C. §§ 703–712; Ch. 128; July 13, 1918; 40 Stat. 755). We, therefore, used MSY as the benchmark and set *F*_0_ = 1 for all populations. We recognize that managing harvest for MSY increases the potential for overharvest in the face of uncertainty and stochasticity, however, we selected *F*_0_ = 1 as a basis for this assessment because no more restrictive objective has been agreed upon by the waterfowl management community.

### Estimating *r*_max_

We defined *r*_max_ as the maximum growth rate achievable by a population when that population is not exposed to the source of mortality of interest (in this case harvest), is not under any resource limitations causing density-dependent regulation, and is experiencing otherwise average environmental conditions. This definition implies that *r*_max_ is not a constant, fixed for either a species or a population, but rather is determined by a species’ life history traits as expressed in a particular environmental setting [21,32,33]. This further implies that *r*_max_ for a population can change over time based on change in the mean environmental conditions experienced by the population, including changes in the degree of anthropogenic factors, besides harvest, affecting mortality or recruitment.

A variety of methods exist to estimate *r*_max_ [21] and each has inherent assumptions and implications with respect to the evaluation of harvest sustainability. Because sea ducks exhibit varying degrees of age structure with respect to reproductive rates [19, 34–38] we used the age-structured population projection matrix **A** (shown below for 3 age classes) [39] to estimate the maximum finite population growth rate in the absence of harvest (*λ*_max_):

$$A=\left[\begin{array}{ccc} b_{1}\times p_{1} & b_{2}\times p_{2} & b_{A}\times p.ad\\ p_{1} & 0 & 0\\ 0 & p_{2} & p.ad \end{array}\right]$$ (3)

Parameters *p*_1_, *p*_2_, and *p*.*ad* represent juvenile (i.e, post-fledging young), 2^nd^ year subadult, and adult age class survival rates in the absence of harvest, respectively, and *b* represents fecundity (fledged juveniles per adult) for the same age classes, at low density.

We used a post-birth pulse formulation of the projection matrix with 4 age classes (juveniles, 2^nd^ year and 3^rd^ year subadults, and adults) for eider [36] and 3 age classes (juveniles, 2^nd^ year subadults, and adults) for the other populations [34, 35, 37, 38] to allow for age-specific fecundity and survival. We calculated *λ*_max_ as the dominant eigenvalue from the projection matrix using the *popbio* package in program R [40, 41] and estimated *r*_max_ as *λ*_max_− 1 for a discrete growth process.

While the projection matrix formulation requires the estimation of a larger number of input parameters than some other methods of estimating *r*_max_ (i.e., demographic invariant method, DIM) [42], it has the advantage of greater generality and flexibility from a management perspective. Specifically, the projection matrix incorporates both fecundity and survival processes, which is important since the maximum values of both are affected by environmental conditions and anthropogenic stressors that constrain maximum growth for a population.

Although context- or population-specific *r*_max_ estimates are of greatest utility in harvest management, the challenges in estimating demographic rates under conditions applicable to *r*_max_ led us to also use the DIM to generate alternative estimates of *r*_max_. The DIM requires only estimates of adult survival and age-at-first-breeding, and is based on relationships between survival, fecundity, and generation time that apply broadly within taxonomic groups. We computed DIM estimates of *r*_max_ using (Eq 15 in [42]) as:

$$r_{max}^{DIM}\approx \lambda_{max}^{DIM}-1\approx \frac{(p.ad\times \alpha -p.ad+\alpha -1)+\sqrt{{\left(p.ad-p.ad\times \alpha -\alpha -q\right)}^{2}-4\times p.ad{\times \alpha }^{2}}}{2\times \alpha }-1$$ (4)

where *p*.*ad* is adult survival and *α* is age at first breeding, both measured under optimal growth conditions. We consider DIM estimates of *r*_max_ as theoretical maximum values for each species and contrasted them to the population-specific estimates derived from the matrix models, though we did not apply DIM estimates in the harvest assessment or comparisons.

We contrasted the DIM and projection matrix estimates of *r*_max_ by examining overlap in the computed credible intervals for allowable take. To apply the DIM, we computed mean body mass of adult females from published estimates [34, 37, 38, 43–48] and used an allometric relationship between body mass and adult survival (Eq 21 in [22]) to compute adult survival in the absence of harvest under ideal growth conditions as:

$$p.ad=p^{1/(\exp\left[3.22+0.24\times \log\left(M\right)+e\right]-1)}$$ (5)

where *p*.*ad* is adult survival, *M* is adult female body mass (kg), *p* ~ beta(3.34, 101.24) and *e* ~ Normal(0, σ^2^ = 0.087).

### Developing probability distributions for demographic parameters

We relied on a combination of published and unpublished data and estimates as well as the results of a formal expert elicitation to specify probability distributions for parameters. We sought to develop probability distributions that reflected uncertainty about the true mean value of each demographic parameter for each population.

#### Literature search

With the aid of the Sea Duck Joint Venture (SDJV) Continental Technical Team, we searched both published and “gray” literature and compiled information on survival rates, fecundity components, overall fecundity, age of first and last breeding, population size, and harvest rates and/or total harvest for the populations under consideration. The SDJV is a U.S.–Canadian partnership formed under the North American Waterfowl Management Plan to address information deficiencies and improve sea duck management. Sea duck researchers and managers participating in the SDJV were organized into species committees to assist in the literature search. Committees were provided forms to aid in the literature search which specified parameters of interest. No other sideboards were provided for the literature review.

We sought estimates of the mean values of these parameters for each of the populations of interest under conditions corresponding to maximum growth potential. In comparison to other waterfowl species, available information on demographic rates for sea ducks is limited in both spatial and temporal scope. For many parameters, published demographic rates were unavailable, available only from dated studies, or applicable only to local study populations (S1 Table). Vital rates presented typically applied to populations subjected to harvest and possibly experiencing density-dependent regulation and, therefore, were not directly applicable in estimating *r*_max_. Moreover, we were concerned that estimates of statistical variability associated with published demographic rates for local populations were not representative of the uncertainty about the true population means for those parameters.

The paucity of available data led us to also conduct an expert elicitation process to supplement available information on demographic rates and their uncertainty. In deciding what published or unpublished data to use directly in the assessment, we attempted to select only estimates that were of the parameter of interest, not requiring major assumptions or adjustments. We also sought estimates that provided associated measures of variation or where such measures could be derived from the data presented. Other relevant data, that did not meet these criteria, were provided to the expert panel to assist in formulating the experts’ input.

Because most available sea duck population estimates [11, 49] did not incorporate methods to adjust for various observer-biases, such as detection bias, and were not collected at the time of year required by the assessment, no population data were used directly in the assessment, rather we relied solely on the values provided through the elicitation. Similarly, survival estimates tended to be for the adult male cohort and for populations undergoing harvest. Total harvest estimates, though derived partly from estimates from statistically rigorous national harvest surveys, included uncertain and incomplete estimates of subsistence harvest. Because of these limitations, survival and harvest data used in the assessment were also derived solely from expert judgments that were informed by available data. For a number of fecundity parameters we were able to find estimates meeting our criteria. Since it was not possible to determine if those estimates were obtained while density regulation was occurring, we chose to incorporate those estimates directly in the assessment along with the elicitation results. More detail on data used in this assessment is found in subsequent sections.

#### Expert elicitation process

Increasingly, researchers have employed formal methods of elicitation to synthesize expert judgments about uncertain quantities or processes (e.g., [50–52]). Elicitation refers to formal methods for estimating parameters when no empirical estimates exist on the basis of limited data and expert knowledge and opinion. We used expert elicitation to generate probability distributions for age-specific survival and fecundity parameters in the absence of harvest and at low density, fall population size, observed sport and subsistence harvest, and differential vulnerability (*DV*) of age classes to harvest. We summarized the information for each species obtained through the literature search (S1 Table) and provided this to an expert panel of sea duck ecologists and managers in the U.S. and Canada as background information for the elicitation. The panel was selected based on previous research or management experience with sea ducks and on willingness to participate. We requested that each panel member identify any additional published or unpublished data sources not summarized in the literature review. We also provided each panel member with an elicitation form and requested that they provide four values for each parameter (Table A in S2 File) in accordance with the four-point elicitation method described by [53]: the experts’ best appraisal of the true population mean, the highest the mean could be, the lowest the mean could be, and a probability reflecting their confidence that the true mean was within the bounds they specified. Panel members were not required to specify values for all parameters and only offered judgments about select parameters at their discretion. We reviewed responses from each expert and when we perceived ambiguity in a panel member’s response, we sought clarification. We then compiled input from all experts and returned the compilation to them for review, without revealing the names of the panel members. Experts were asked to view their peers’ responses, identify areas of concern or misunderstanding, and revise their own values as they deemed appropriate.

#### Combining sources of information and generating probability distributions

We used the results from the elicitation to construct expert-specific probability distributions using the methods described by ([54], p. 186). We also described a probability distribution for individual parameters from literature values when sufficient published and unpublished information existed (Table A in S2 File). We generated beta distributions for binomial parameters (e.g., survival, nest success, hatch success, etc.) with values constrained between 0 and 1. For other parameters, whose values were constrained to be > 0 we used the lognormal distribution to describe uncertainty. In the case of breeding propensity, where experts provided a best estimate but no measure of uncertainty, we assumed a binomial variance for all species where only 2 age class-specific propensities were estimated, and a multinomial variance for eider for which propensity differed among 2^nd^ and 3^rd^ year subadults and adults. We used the *qmedist* function of the *fitdistplus* package in R to derive distribution parameters and then functions *rbeta*, *rlnorm*, or *rbinom* to generate probability distributions for the simulation [55]. We used Monte Carlo simulation (10,000 samples) to select randomly, and with equal probability, from the probability distributions derived from expert responses as well as available published or unpublished data, in order to develop a single probability distribution characterizing uncertainty for each parameter across all experts and data sources.

We quantified uncertainty for each parameter distribution by calculating a CV for non-binomial parameters and concentration for binomial parameters. Concentration is a measure of relative proportional uncertainty for binomials and was computed as:

$$Conc=\frac{SD}{\sqrt{p(1-p)}}$$ (6)

where SD is the standard deviation and *p* is the median simulated value of the parameter [56].

#### Parameters

***Survival (p1*, *p2*, *p3*, *p*.*ad)*.** To obtain survival rates, we compiled literature values from mark–recapture studies or banding data. Literature values generally represented survival of adults of populations subjected to harvest. Therefore, we also modeled female survival estimates based on an allometric relationship predicting maximum survival as a function of body weight (Table A in S2 File; [22]). Both literature-derived estimates and those based on the allometric relationship [22] were provided to the elicitation panel as aids in specifying values for mean juvenile, subadult, and adult survival rates in the absence of harvest. The number of subadult age classes differed for each population depending on published information and expert opinion regarding the breeding propensity of each age class.

***Fecundity (b)*.** Fecundity was defined as the number of female offspring fledged per reproductive female per year. To estimate *r*_max_, reproductive rates should be representative of a population undergoing unrestrained growth, without density-dependent regulation, under average environmental conditions [21]. We estimated fecundity using two methods: (1) as the product of breeding propensity, clutch size, nest success, hatch success, duckling survival, and duckling sex ratio, and (2) from unadjusted female harvest age ratios (*i*.*e*, first-year females/adult females) derived from waterfowl parts collection surveys in the U.S. and Canada [57], divided by differential vulnerability (*DV*). Differential vulnerability estimates the extent to which juvenile birds are more vulnerable to hunting than adults; it is computed by dividing the band recovery rate of juvenile females by that of adult females. We computed variance of unadjusted age ratio by summing the number of juvenile and adult females wings received in parts collection surveys from 2004–2013 (Table B in S2 File). Treating the number of juvenile female wings as a random binomial variable, we simulated binomial proportions from the number of juvenile and total juvenile and adult wings, then converted proportions back to age ratios. Wing samples for western black scoter were insufficient and we did not include unadjusted age ratio or *DV* in the analysis for this population. Conceptually, the age-ratio based estimates, if unbiased, should be viewed as minimum estimates of fecundity at maximum growth rate since they were likely not derived when populations were at low density. However, we chose to include, and equally weight, both methods of estimating fecundity because of challenges in estimating individual reproductive rates under optimal growth conditions (i.e., *r*_max_) and uncertainty about the degree to which density dependent regulation of fecundity was occurring in each population. For all populations except white-winged scoter and long-tailed duck, we created the combined fecundity distribution by sampling from the distributions for the 2 independent methods with equal probability. For white-winged scoters and long-tailed ducks, the 2 independent methods of estimating fecundity produced widely divergent values and the combined distribution produced by these methods was bimodal. Since the bimodality was an artifact of differences in estimation methods and not of a hypothesized biological mechanism, we generated unimodal fecundity distributions for these populations by combining all simulated values of fecundity from both methods, computing mean and variance, and using those statistics to generate a single combined lognormal distribution. While the combined fecundity distribution of common eider was weakly bimodal, in this case the pattern resulted from a hypothesized mechanism of ‘boom and bust’ reproduction (e.g., see p. 245 of [58]) and, so, was retained in the final combined distribution.

***Population size (N*).** With the exception of a survey of breeding western black scoters that covered >80% of the population’s breeding range and was conducted annually from 2004–2012 [11], sea duck abundance monitoring programs are poor. At the time that the principal duck breeding population surveys [59] were developed in North America in the 1950s and 1960s, sea ducks were not considered species of socio-economic importance. Surveys did not include significant portions of the breeding ranges of some sea duck species, particularly portions of the boreal forest and tundra where operations were logistically challenging and costly. In addition, certain species such as scoters were aggregated in breeding population counts, precluding estimation of species-specific abundance. While expansion of breeding population surveys has occurred since [11], limitations of these surveys led managers to recently conduct comprehensive surveys of certain wintering sea duck populations [11,49,60]. Winter surveys have additional challenges associated with aggregated and temporally-variable bird distributions as well as greater risk to survey personnel safety associated with offshore operations [49, 60]. Despite the challenges encountered in winter surveys, the insufficient spatial coverage of breeding surveys and/or lack of species-level breeding population data for our species of interest led us to provide population estimates from winter surveys to the expert elicitation panel.

Winter abundance data for eastern and western surf scoters, eastern black scoter, white-winged scoter, and long-tailed duck were obtained through recently conducted winter surveys of the Atlantic and Pacific Coasts and Great Lakes ([49]; Sea Duck Joint Venture, unpublished data). Winter abundance estimates of western black scoters from Pacific Coast winter surveys were credible when compared with the breeding population survey estimate, considering that the winter estimate includes young-of-the-year birds [11].

To use winter population estimates in the PTL framework, we had to either: 1) convert the estimates to breeding population (pre-birth pulse) estimates, or 2) convert them to fall population (i.e., post-birth pulse) estimates and modify the PTL framework to accommodate a post-birth pulse estimate. We chose to modify the PTL framework to accommodate fall population estimates because we believed that there were fewer assumptions in converting winter population estimates to the preceding fall population than to estimates of spring breeding populations. To accommodate our post-growth formulation of the PTL framework, winter population estimates were converted to estimates for the preceding fall by adding the estimates of retrieved and unretrieved harvest, assuming negligible natural mortality. Converting winter population estimates to the preceding spring would have also required assumptions about mortality during the breeding season. Converting winter estimates to breeding population estimates by projecting forward would require assumptions about mortality from mid-winter through spring migration during a period of more extreme environmental conditions and possibly greater resource limitation. We provided winter abundance estimates, which were uncorrected for detection bias, as well as estimates of detection rate during winter aerial surveys [J. Leirness, U.S. Fish and Wildlife Service, unpublished data] to the expert elicitation panel as background information in formulating their best estimates of fall population size.

***Harvest and harvest rate (H*.*obs*, *h*.*obs)*.** Harvest estimates utilized in this assessment consisted of both fall and winter sport harvest and subsistence harvest for scoters and long-tailed ducks. We compiled estimates of fall and winter sport harvest obtained from the national harvest surveys that are conducted annually in the U.S. and Canada. We represented fall and winter sport harvest (*H*_Sp_) as the 2004–2013 mean of the combined estimated harvest in the U.S. and Canada (Table C in S2 File), and multiplied that mean by 0.731 (see [18, 61]) to account for a presumed bias in harvest estimates that may be related to prestige, non-response, or other factors associated with the national harvest surveys.

For subsistence harvest (*H*_Su_, we used estimates for Alaska for 2011 [60], as well as subsistence harvest estimates obtained from several one-time surveys of aboriginal peoples that were conducted in specific regions across Canada from the 1980s to the 2010s ([18,62,63]; Cree Trappers Association, Cree Regional Authority, and; Canadian Wildlife Service, unpublished data; C. Lepage, Canadian Wildlife Service, personal communication). Hunting, whether for sport or subsistence, includes associated unretrieved kill (*i*.*e*., crippling loss), which is assumed to be about 30% of retrieved harvest of sea ducks [18]. We adjusted the combined total harvest estimates, including both sport and subsistence, (*H*_SS_) for crippling loss (*C*) to arrive at total observed harvest (*H*.*obs*):

$$H.obs=\frac{H_{SS}}{\left(1-C\right)}$$ (7)

Because one co-author had extensive experience estimating North American waterfowl harvest, we did not use expert panel elicitation responses to develop probability distributions to characterize uncertainty in observed harvest but, rather, used elicitation results provided by this co-author (Table D in S2 File).

An estimate of the total harvest (in numbers of birds) of American common eiders was not needed because harvest rates since 2002 have been estimated from banding data for this population. We used banding and dead recovery data obtained from the Bird Banding Laboratory (Table E in S2 File) and the Brownie et al. dead recovery model [31] to estimate recovery rates for nesting and molting female birds. The dead recovery model assumes that no mortality occurs between banding and exposure to harvest. Molting birds are likely to meet this assumption, but we were concerned that the molting birds might underrepresent breeding females. Therefore, we included nesting birds in the recovery analysis. We acknowledge that some mortality occurred between nesting and the hunting season; however, given the high annual survival rate of female eiders [64], we assume that any violation of this assumption would lead to only a slight underestimate of recovery rates. We converted recovery rates to overall rates of retrieved and unretrieved harvest, *h*.*obs*, by dividing the recovery probabilities by a reporting probability estimated for mallards in the Atlantic Flyway [30] and by crippling loss. We incorporated the uncertainty in recovery rates, reporting rates, and crippling loss into probability distributions describing uncertainty in overall rates of retrieved and unretrieved harvest.

***Functional form of density dependence (θ)*.** In the theta-logistic growth model, *θ* defines the functional form of density dependence [29], with *θ* = 1 reflective of linear density dependence, *θ* < 1 consistent with density dependence that is strongest when population size is far from *K* (i.e., carrying capacity), and *θ* >1 consistent with density dependence that is strongest when a population is close to *K*. Values of *θ* < 1 are typical of fast-growth (i.e., *r*-selected) species while *θ* > 1 are typical for slow-growth or *K*-selected species [29]. The form of density dependent response has consequences for estimates of allowable harvest with allowable harvest estimates for *θ* >1 exceeding those for *θ* ≤ 1. We were unable to find any empirical estimates of *θ* for sea ducks in the literature. We considered using elicitation to derive estimates of *θ*, however, felt that it would be extremely difficult for the expert panel members to provide educated opinions on this parameter. We, therefore, selected a published value of *θ* for a North American goose species (snow goose, *Anser caerulescens*, *θ* = 2.05) [65] which, based on similarity in life history, we felt could reasonably be used to bracket values of *θ* between 1 and 2. We did not attempt to fit a probability distribution for *θ* or subject it to sensitivity analysis similar to the other parameters, instead ran 2 independent simulations, one where *θ* = 1 and another where *θ* = 2, and contrasted estimates of allowable harvest and risk of overharvest.

### Comparison of allowable harvest to observed harvest

Using Monte Carlo simulation, we drew 10,000 samples from the probability distributions described for the demographic parameters and computed an allowable harvest estimate for each sample [8, 22]. During each simulation, we also sampled from the probability distribution for total observed harvest (for scoters and long-tailed ducks) and observed harvest rate (for eiders). The final step of each simulation was to compare the resultant allowable and observed harvest values. We then computed the proportion of iterations where observed harvest (or harvest rate) was less than allowable harvest (or harvest rate). We interpreted those proportions as measures of the risk of harvesting in excess of MSY, accounting for uncertainty in both allowable and observed harvest. Proportions closer to zero reflected a higher risk of overharvest, given the benchmark of MSY (i.e., *F*_0_ = 1). We repeated the simulation twice, once with *θ* = 1 and once with *θ* = 2, and summarized results independently.

### Sensitivity of harvest comparisons and influential parameters

We conducted a sensitivity analysis to identify the individual demographic parameters whose uncertainty most influenced inferences about the risk of overharvest. Within the PTL framework, uncertainty in a given parameter will most greatly influence these inferences if *r*_max_ (and hence allowable harvest) is highly sensitive to the parameter, and if there is a large degree of uncertainty about the true parameter mean.

We compared the sensitivity of the difference between allowable and observed harvest estimates (or harvest rate for eiders) to uncertainty in the component parameters of *r*_max_, fall population size, and observed harvest by comparing the slopes of the linear relationships between the simulated differences in allowable and observed harvest and simulated values of each parameter. So that the slopes were directly comparable, we first standardized values of the probability distribution for each demographic parameter. We used the *lm* function in R to fit the linear model used to assess sensitivity as:

$$H_{diff}=b_{0}+b_{1}x^{std}$$ (8)

where *H*_diff_ are the values of allowable harvest minus observed harvest (or harvest rate for eiders), *b*_0_ is the intercept, *b*_1_ the slope, and *x*^std^ are the standardized values of the parameter of interest. The slopes (*b*_1_) represent the sensitivity of the risk of overharvest to uncertainty in the component parameters. Parameters to which the risk of overharvest is highly sensitive have a high degree of uncertainty, a large influence on *r*_max_, or both.

## Results

### Elicitation

We asked 38 North American sea duck researchers and managers for their input. Nineteen experts responded, of whom 14 provided input for at least one population. We received input from 4 experts for American common eider, eastern black and surf scoters, and long-tailed duck; 3 experts for western surf scoter; 2 experts for western black scoter; and 1 expert for white-winged scoter. The number of experts providing information for each population and parameter is summarized in Table F in S2 File, along with their responses. Data compiled from the literature were usually similar to the values elicited through expert judgment (i.e., intervals submitted by expert judgment overlapped the values from the literature), with a few exceptions (Table F in S2 File). Hatch success for common eider based on the literature was less than the lowest value submitted by all three experts who provided input on that demographic rate.

### Parameter distributions

**Fig 2:**
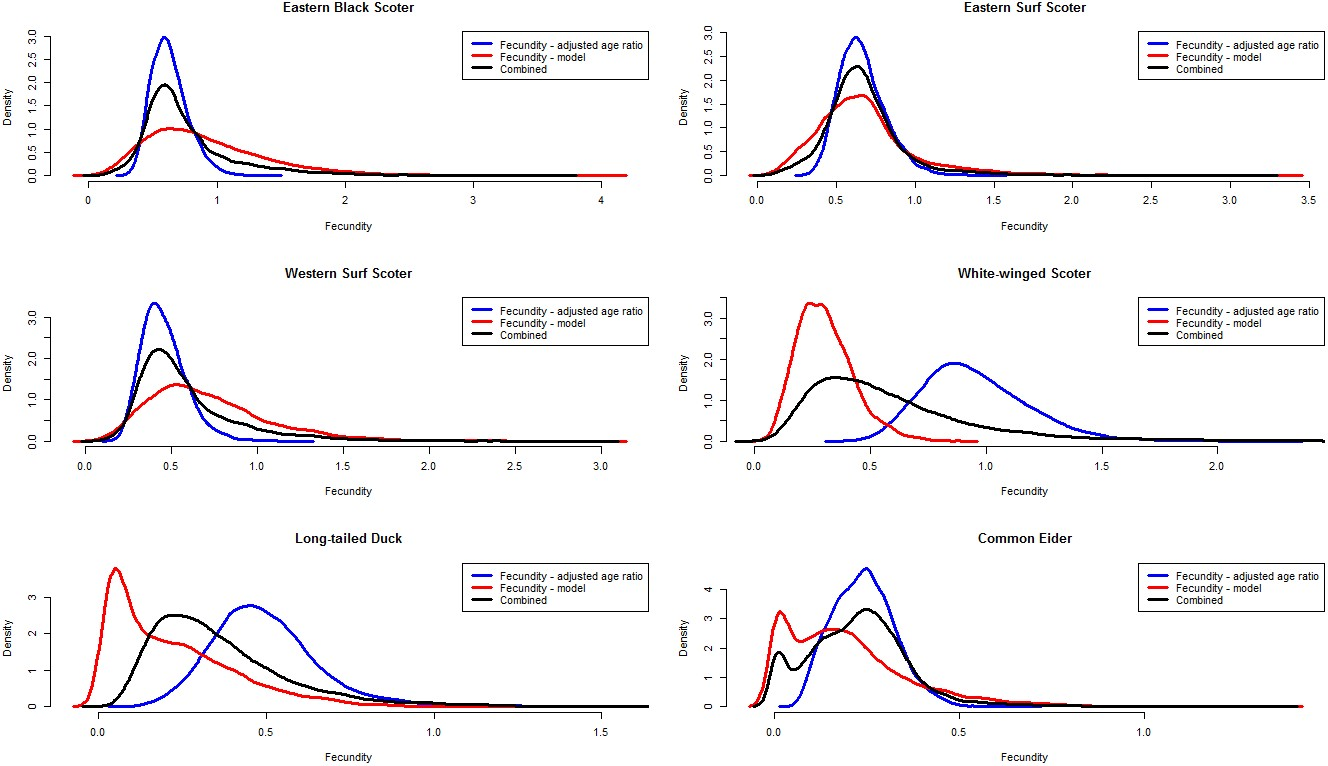
Probability distributions depicting uncertainty in two alternative methods of estimating fecundity for seven populations of North American sea ducks as well as combined probability distributions that weight both methods equally.

Expert judgment and data from the literature indicated that breeding propensity was approximately 25% (range = 21%-28%) for 2^nd^ year subadults and 90% (range = 85%-94%) for adults in the species we considered (Table 1). Median clutch size ranged from 7 to 9 for scoters and long-tailed duck and was about half that for common eider (Table 1). Nest success estimates varied nearly by a factor of 3 among species, ranging from 24% for white-winged scoter to 68% for common eider. Hatch success was high (>86%) for all species except long-tailed duck, which had an estimate of 73% (Table 1). Like nest success, duckling survival varied widely among species ranging from 17% for common eider to 45% for eastern black scoter. Combining these components into overall fecundity indicated that productivity (fledged juvenile female/adult female) of common eider (median = 0.17), long-tailed duck (0.18), and white-winged scoter (0.29) was lower than it was for black and surf scoters (0.64–0.82; Fig 2, red lines). The fecundity estimates based on age ratios corrected for *DV* were similar to the estimates based on reproductive components for eastern surf scoter; approximately 30% lower for eastern black and western surf scoters; and substantially higher (approximately 200%) for white-winged scoter and long-tailed duck (Fig 2, blue lines). Fecundity based on age ratios was also 71% higher for common eider, but still low compared to all other species (Fig 2, blue line).

**Table 1:** Median and 95% credible intervals of final probability distributions for parameters used in the harvest potential assessment of seven sea duck populations, based on available empirical data and expert elicitation.

Parameter	Common Eider	Eastern Black Scoter	Eastern Surf Scoter	Western Black Scoter	Western Surf Scoter	White-winged Scoter	Long-tailed Duck
Breeding propensity of females at age 2 (*bp2*)	0.21 (0.13,0.30)	0.24 (0.13,0.43)	0.24 (0.13,0.43)	0.25 (0.17,0.34)	0.22 (0.14,0.33)	0.28 (0.12,0.48)	0.28 (0.11,0.43)
Breeding propensity of females at age 3 (*bp3*)	0.75 (0.60,1.00)	na	na	na	na	na	na
Breeding propensity adult females (*bp*.*ad*)	0.92 (0.60,1.00)	0.92 (0.73,0.99)	0.93 (0.73,0.99)	0.91 (0.67,0.99)	0.85 (0.65,0.99)	0.94 (0.77,0.99)	0.88 (0.63,0.99)
Clutch size (cs)	3.96 (2.93,5.23)	8.02(5.65,10.37)	7.45 (5.95,9.77)	7.91 (5.90,10.09)	7.24 (3.94,10.38)	8.81 (7.41,10.47)	7.05 (5.16,8.77)
Nest success (*ns*)	0.68 (0.40,0.88)	0.63 (0.36,0.93)	0.53 (0.30,0.89)	0.48 (0.18,0.93)	0.66 (0.34,0.91)	0.24 (0.10,0.43)	0.46 (0.18,0.70)
Hatching success (*hs*)	0.86 (0.56,0.95)	0.92 (0.60,1.00)	0.94 (0.58,1.00)	0.97 (0.89,1.00)	0.97 (0.49,1.00)	0.86 (0.83,0.90)	0.73 (0.19,0.94)
Duckling survival (*ds*)	0.17 (0.00,0.53)	0.45 (0.17,0.75)	0.42 (0.18,0.65)	0.40 (0.20,0.76)	0.40 (0.21,0.62)	0.36 (0.27,0.45)	0.24 (0.07,0.49)
Ratio of juvenile to adult hen wings submitted and collected in the US and Canadian national harvest survey (*wings*)	0.58 (0.47,0.70)	1.42 (1.18,1.72)	1.49 (1.27,1.76)	NA	0.96 (0.70,1.31)	2.14 (1.73,2.71)	1.25 (1.08,1.45)
Differential vulnerability; vulnerability of juvenile to adult females to harvest (*DV*)	2.39 (1.44,5.74)	2.31 (1.55,3.41)	2.31 (1.55,3.41)	NA	2.18 (1.41,3.37)	2.31 (1.55,3.41)	2.61 (1.42,5.32)
Juvenile, first-year survival (*p*1)	0.65 (0.40,0.85)	0.67 (0.50,0.80)	0.66 (0.50,0.80)	0.66 (0.50,0.80)	0.58 (0.29,0.81)	0.67 (0.58,0.75)	0.63 (0.45,0.78)
Subadult, 2nd year survival (*p*2)	0.89 (0.79,0.96)	0.82 (0.71,0.95)	0.82 (0.71,0.95)	0.82 (0.70,0.95)	0.88 (0.65,0.97)	0.75 (0.66,0.83)	0.71 (0.47,0.85)
Adult survival (*p*.*ad*)	0.90 (0.78,0.96)	0.88 (0.79,0.95)	0.88 (0.79,0.95)	0.88 (0.79,0.95)	0.88 (0.65,0.97)	0.84 (0.75,0.90)	0.81 (0.58,0.91)
Fall population size; post-birth pulse population size (*N*^FF^)	na	464132 (272345,770082)	387514 (150571,890743)	218541 (186578, 256323)	413687 (211809, 888205)	536077 (382127,754206)	777748 (517211,1207488)
Observed total sport and subsistence harvest unadjusted for crippling loss; for COEI this is observed harvest rate unadjusted for crippling loss (*H*.*obs; h*.*obs*)	0.018 (0.016,0.021)	19915 (14196,28221)	26227 (20265,34208)	11705 (7503,18130)	6688 (3885,11493)	16549 (10246,27113)	29996 (24015,37716)
Crippling loss or unretrieved harvest (*crip*)	0.30 (0.18,0.43)	0.30 (0.19,0.43)	0.30 (0.19,0.43)	0.30 (0.18,0.43)	0.30 (0.18,0.43)	0.30 (0.18,0.43)	0.30 (0.19,0.43)

Experts expressed considerable uncertainty in most of the fecundity parameters, particularly in hatch success, nest success, and duckling survival (Table F in S2 File). High uncertainty in the components of fecundity resulted in greater uncertainty in the model-based estimates of fecundity (CV range = 40% for white-winged scoter to 85% for common eider) compared to the estimates based on adjusted age ratios from the wing data (all CV ≤34%).

Estimated adult survival rates in the absence of harvest (and at low density) for common eider (median = 90%) and both populations of black and surf scoters (median = 88%) were similar, whereas the estimates for white-winged scoter (median = 84%) and long-tailed duck (median = 81%) were lower (Table 1). Survival rates for 2nd year subadult birds were similar to adults for common eider and western surf scoter, but between 6 and 12% lower than adults for all other populations. Juvenile survival was 20–34% lower than adult survival for all species (Table 1). Estimates of survival based on the allometric relationship with body weight were similar to those based on expert judgment for common eider (*S*_allometric_ = 0.90, SD = 0.04), black scoter (*S*_allometric_ = 0.88, SD = 0.05), and surf scoter (*S*_allometric_ = 0.87, SD = 0.05), but higher than the experts’ estimates for white-winged scoter (*S*_allometric_ = 0.88, SD = 0.04) and long-tailed duck (*S*_allometric_ = 0.86, SD = 0.05). Relative uncertainty in survival parameters (all <30%) was much lower than fecundity, particularly for adult survival (all ≤25%; S2 Table).

Total observed harvest, unadjusted for crippling loss, ranged from 6,688 per year for western surf scoter to 29,996 per year for long-tailed duck and the CVs decreased with larger harvest estimates (Table 1). Estimated fall population size ranged from about 220,000 for western black scoter to almost 780,000 for long-tailed duck. Uncertainty was relatively high for fall flight estimates for both surf scoter populations, and was lower for all other populations (CV ≤26%; S2 Table).

### Comparisons of allowable and observed harvest

The simulations produced median *r*_max_ estimates ranging from -0.063 for long-tailed duck to 0.143 for eastern black scoter (Table 2). The median estimate of *r*_max_ for long-tailed duck was negative. Median simulated *r*_max_ was far lower for long-tailed duck than for any other population (Table 2). The median *r*_max_ for each population computed from the projection matrix was lower than the maximum theoretical *r*_max_ computed using the DIM; however, the credible intervals of the two estimates overlapped for all populations except long-tailed duck (Table 2).

**Table 2:** Median (and 95% credible intervals) of simulation-derived probability distributions for theoretical maximum values of *r*_max_ derived using the Demographic Invariant Method, population-specific values of *r*_max_ derived using a projection matrix, allowable total harvest (or harvest rate for eider) derived from population-specific *r*_max_ values at *θ* = 1 and *θ* = 2 to bracket the functional form of density dependence, observed total harvest (harvest rate for eider) including sport and subsistence harvest adjusted for crippling loss, and percent of simulations where observed harvest was ≤ allowable harvest when *θ* = 1 and *θ* = 2 for seven populations of North American sea ducks.

Population	Demographic Invariant Method *r*_max_ (95% CI)	Matrix Model *r*_max_ (95% CI)	Allowable harvest θ = 1 (95% CI)	Allowable harvest θ = 2 (95% CI)	Observed harvest (95% CI)	Percent Observed < Allowable *θ* = 1	Percent Observed < Allowable *θ* = 2
Common Eider	0.155 (0.109;0.218)	0.003 (-0.157; 0.144)	0.001 (-0.085; 0.067)	0.002 (-0.117; 0.087)	0.026 (0.021; 0.033)	19%	26%
Eastern Black Scoter	0.176 (0.120; 0.249)	0.143 (0.005; 0.420)	29,946 (988; 90,952)	39,062 (1,316; 115,850)	28,528 (19,567; 42,281)	53%	68%
Western Black Scoter	0.176 (0.120; 0.249)	0.136 (-0.057; 0.450)	13,844 (-6,351; 40,843)	18,077 (-8,549; 51,319)	16,724 (10,324; 27,019)	41%	52%
Eastern Surf Scoter	0.178 (0.123; 0.247)	0.133 (-0.023; 0.333)	23,359 (-4,225; 76,355)	30,497 (-5,656; 98,627)	37,522 (27,666; 52,108)	22%	37%
Western Surf Scoter	0.178 (0.123; 0.247)	0.079 (-0.131; 0.313)	14,721 (-32,348; 82,245)	19,348 (-44,093;105,207)	9,560 (5,399; 16,939)	60%	66%
White-winged Scoter	0.172 (0.119; 0.245)	0.038 (-0.109; 0.268)	9,864 (-32,019; 65,987)	13,068 (-43,540; 84,905)	23,733 (14,140; 39,899)	29%	37%
Long-tailed Duck	0.184 (0.126; 0.258)	-0.063 (-0.283; 0.121)	-23,940 (-118,692; 61,722)	-32,329 (-167,742;80,713)	42,853 (32,657; 57,977)	5%	7%

Allowable harvest distributions were wide, indicative of a large degree of uncertainty for all populations. The median of the allowable harvest distribution where *θ* = 1 was negative for the long-tailed duck (allowable harvest = -23,940; Table 2). The percent of simulations where observed harvest was less than allowable harvest when *θ* = 1 was 5% and 19% for long-tailed duck and common eider, respectively (Table 2). In contrast, for western surf scoter and eastern black scoter, over half of simulations when *θ* = 1 resulted in observed harvest levels equal to or less than allowable harvest (Table 2).

For simulations where *θ* = 2, median values of allowable harvest distributions were again negative for the long-tailed duck (Table 2). The percent of simulations where observed harvest was less than allowable harvest when *θ* = 2 was 7% and 26% for long-tailed duck and common eider, respectively (Table 2). For western surf scoter and eastern black scoter, the proportion of simulations that resulted in observed harvest levels equal to or less than allowable harvest increased to 66% and 68%, respectively, when *θ* = 2 (Table 2).

### Sensitivity of harvest comparisons and parameter uncertainty

**Fig 3:**
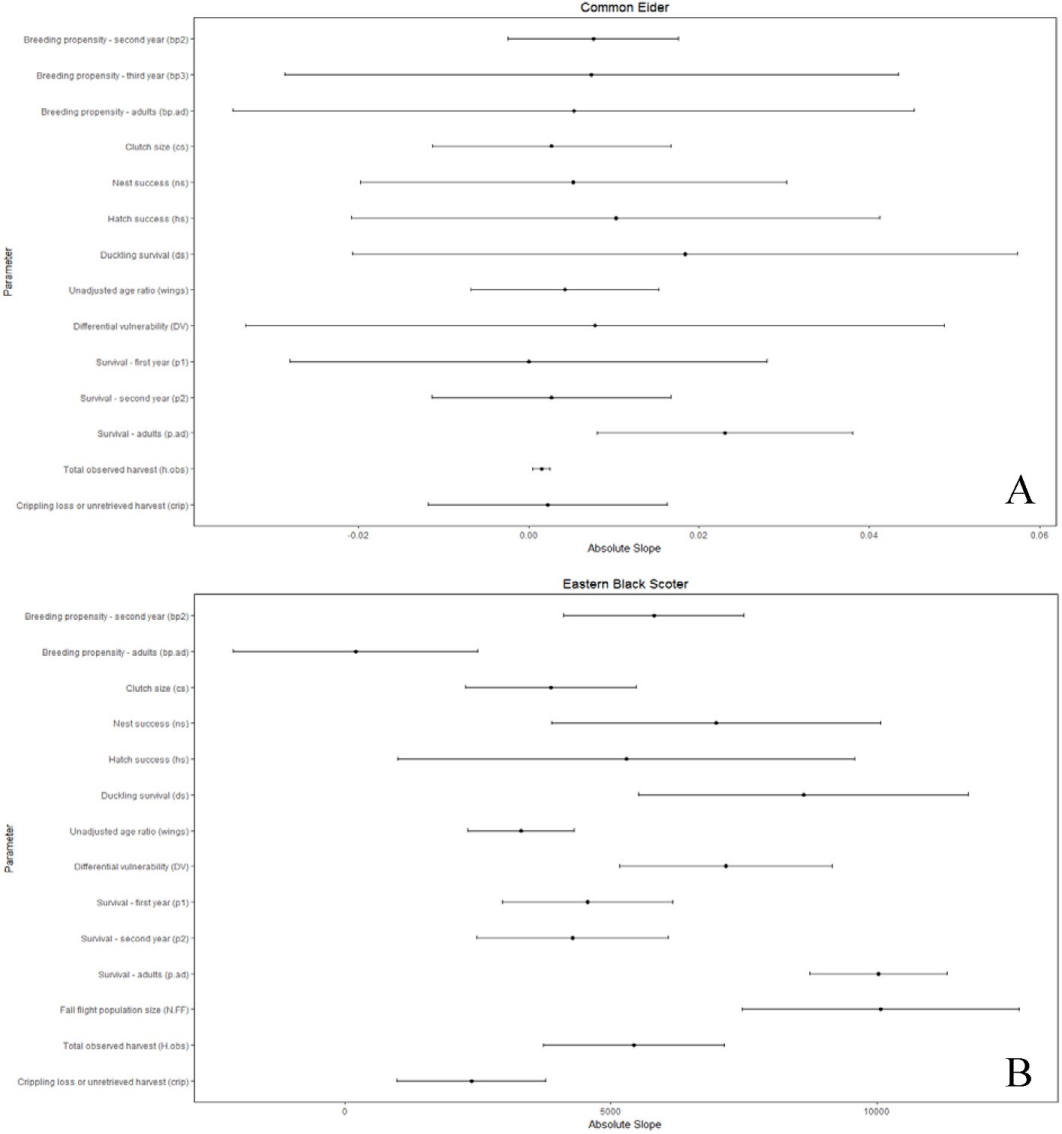
Sensitivity of the difference between allowable and observed harvest to individual demographic parameters as measured by the slope of linear relationships between the harvest difference and standardized values of the demographic parameters, where greater absolute slope indicates higher sensitivity. **Slope is affected by both the inherent sensitivity of growth rate and, hence, allowable harvest, to each parameter and its relative proportional uncertainty. Bars reflecting relative proportional uncertainty represent the coefficient of variation for non-binomial parameters or concentration for binomial parameters and, in both cases, have been multiplied by 100 (divided by 1000 for eiders) for scaling and presentation purposes.** (A) common eider, (B) eastern black scoter.

**Fig 4:**
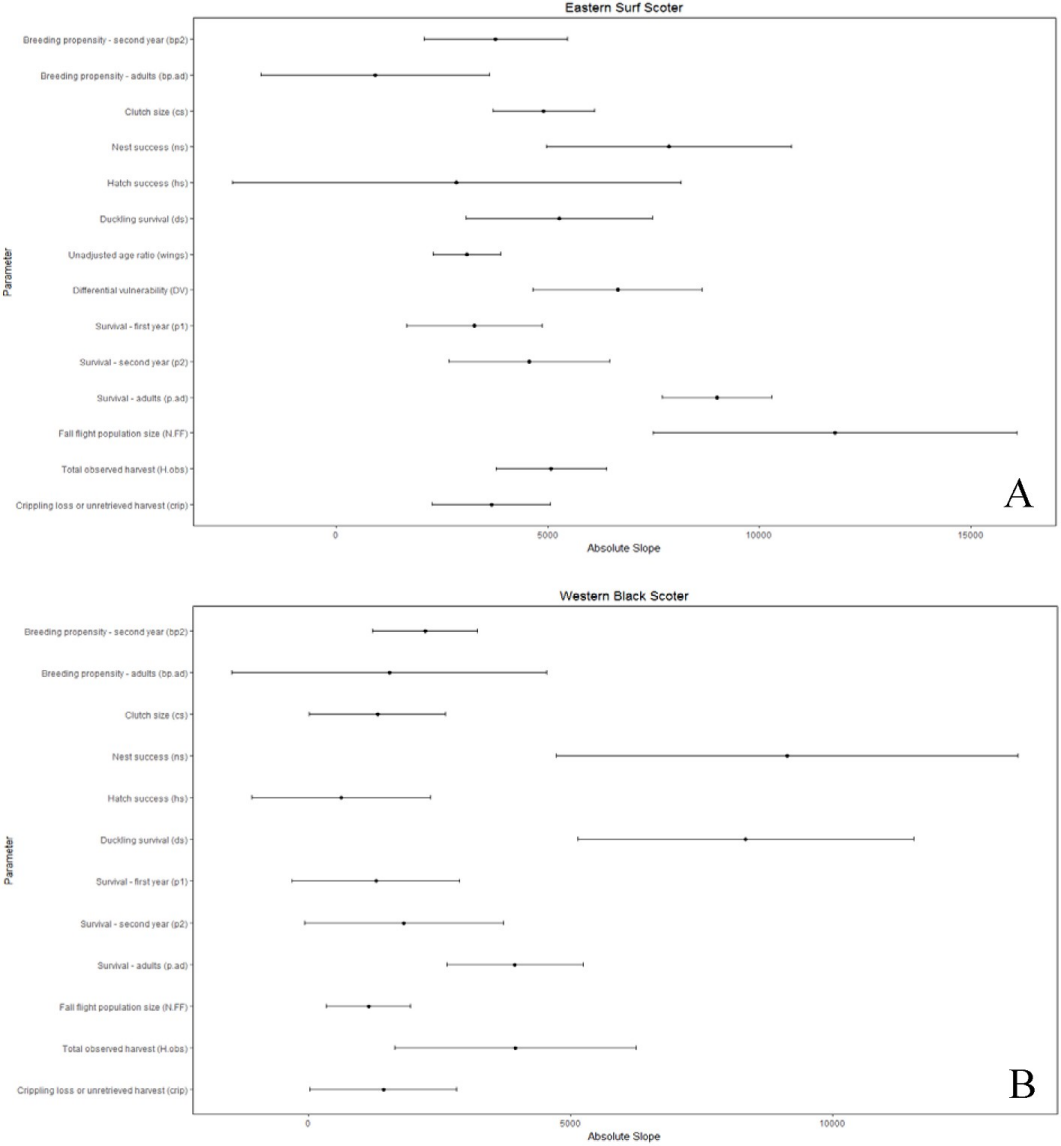
Sensitivity of the difference between allowable and observed harvest to individual demographic parameters as measured by the slope of linear relationships between the harvest difference and standardized values of the demographic parameters, where greater absolute slope indicates higher sensitivity. **Slope is affected by both the inherent sensitivity of growth rate and, hence, allowable harvest, to each parameter and its relative proportional uncertainty. Bars reflecting relative proportional uncertainty represent the coefficient of variation for non-binomial parameters or concentration for binomial parameters and, in both cases, have been multiplied by 100 (divided by 1000 for eiders) for scaling and presentation purposes.** (A) eastern surf scoter, (B) western black scoter.

**Fig 5:**
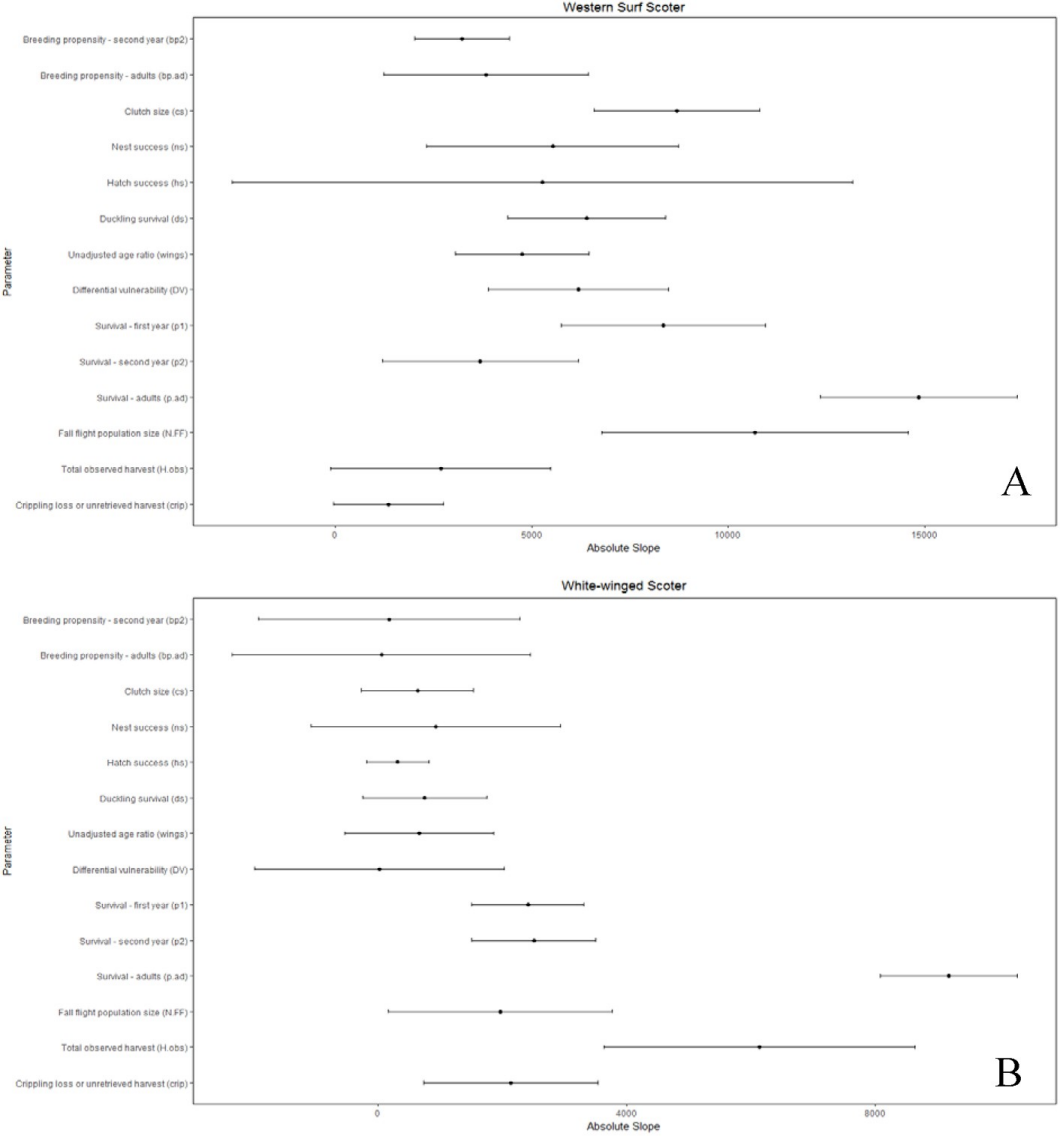
Sensitivity of the difference between allowable and observed harvest to individual demographic parameters as measured by the slope of linear relationships between the harvest difference and standardized values of the demographic parameters, where greater absolute slope indicates higher sensitivity. **Slope is affected by both the inherent sensitivity of growth rate and, hence, allowable harvest, to each parameter and its relative proportional uncertainty. Bars reflecting relative proportional uncertainty represent the coefficient of variation for non-binomial parameters or concentration for binomial parameters and, in both cases, have been multiplied by 100 (divided by 1000 for eiders) for scaling and presentation purposes.** (A) western surf scoter, (B) white-winged scoter.

**Fig 6:**
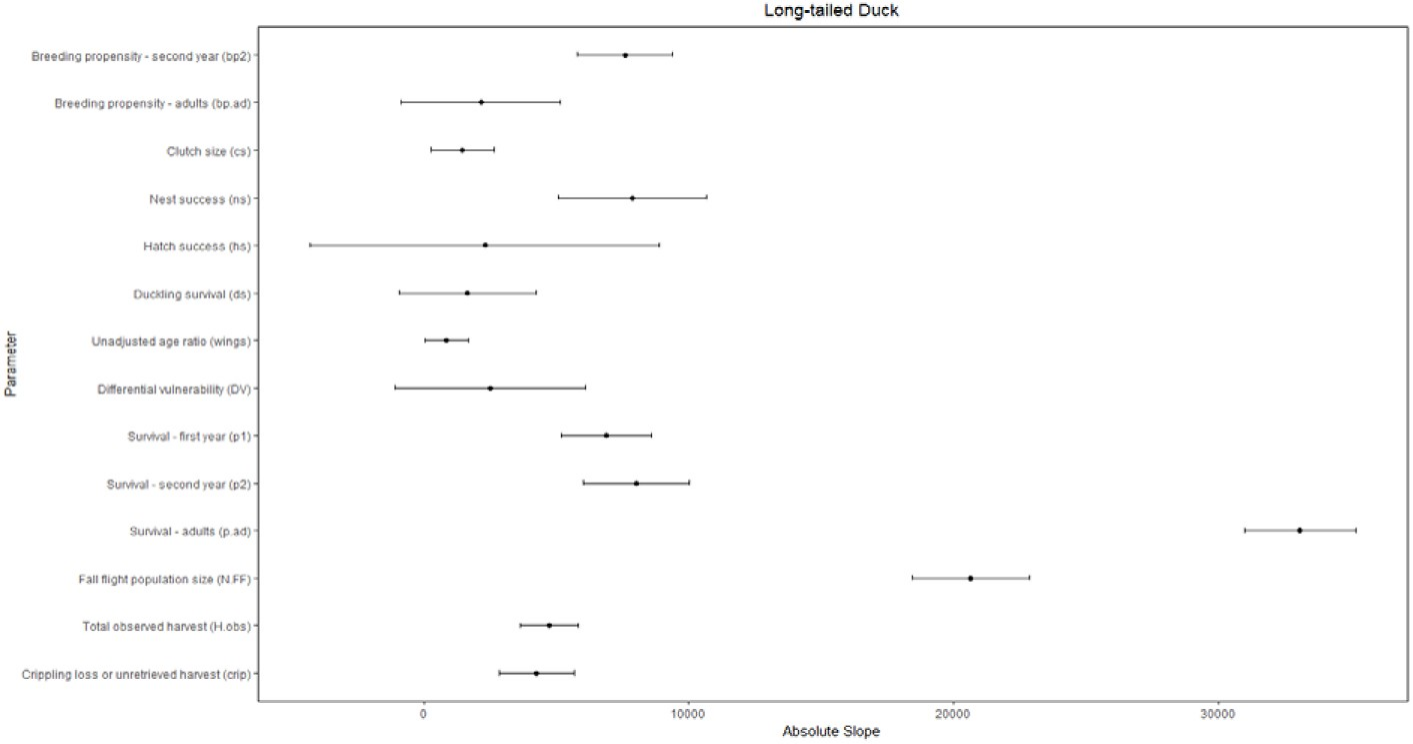
Sensitivity of the difference between allowable and observed harvest of long-tailed duck to individual demographic parameters as measured by the slope of linear relationships between the harvest difference and standardized values of the demographic parameters, where greater absolute slope indicates higher sensitivity. Slope is affected by both the inherent sensitivity of growth rate and, hence, allowable harvest, to each parameter and its relative proportional uncertainty. Bars reflecting relative proportional uncertainty represent the coefficient of variation for non-binomial parameters or concentration for binomial parameters and, in both cases, have been multiplied by 100 (divided by 1000 for eiders) for scaling and presentation purposes.

We considered uncertainty in demographic rates to be important if the variation in the parameters strongly influenced the comparison of allowable and observed harvest. We present only the conclusions of the sensitivity analysis where *θ* = 1, since the conclusions for *θ* = 2 were identical. The composite estimate of fecundity was more important (*b*.*tot*; S2 Table) than any individual parameter for all populations except long-tailed duck, for which it was second in importance (S2 Table). Additionally, the composite fecundity estimates had greater relative uncertainty than most individual parameters (S2 Table). The importance of the individual parameters used to calculate composite fecundity estimates varied by population, but nest success and duckling survival each had a large influence on the composite fecundity estimates for over half the populations, and consequently on comparisons of allowable and observed harvest (Figs 3–6 and S2 Table).

Adult survival was an influential parameter for all populations (Figs 3–6). However, compared to other parameters there was low or moderate uncertainty around estimates of adult survival (all concentrations ≤25%; Figs 3–6). Fall population size estimates were uncertain (CV 18–43%) and highly influential in the sensitivity analysis for most populations. Exceptions were western black scoter (CV = 8%; Fig 4B), where multiple sources of population survey data could be compared, and common eider where population size did not factor into the assessment (Fig 3A).

## Discussion

The few empirical studies that provided demographic information for the species and populations under consideration were typically local in scale and likely were not representative at the population scale. Breeding ranges of North American scoter species and long-tailed duck have only recently been delineated adequately through satellite telemetry projects (e.g., the Atlantic and Great Lakes sea duck migration study; http://seaduckjv.org/science-resources/atlantic-and-great-lakes-sea-duck-migration-study/), and the telemetry data indicate that much of the breeding range of these species lies outside of areas that are currently surveyed annually to estimate breeding waterfowl abundance in North America [11]. Those data also illustrate that reproductive parameter estimates for scoters and long-tailed duck are absent across the great majority of their breeding ranges. Consequently, empirical estimates that were available to us did not necessarily represent population-wide demographics, nor did they adequately characterize the uncertainty around those parameters.

Our effort to augment the empirical data by eliciting judgments from subject-matter experts was useful, despite the low number of responses for individual populations, and we would recommend the practice, especially in data poor applications. North America has few sea duck experts, owing to the difficulties in studying these species in remote areas, and although we solicited input from most of them, several were unable to participate and others were reluctant to provide an estimate for certain parameters, even though the elicitation process allowed them to express their lack of confidence. Most of the experts who did respond only provided estimates for a subset of parameters. Nonetheless, we believe that including the experts’ responses resulted in probability distributions for parameters that better represented uncertainty. Combining the elicited data and limited available empirical data also ensured that the probability distributions reflected parameter values that were presumed representative of the population.

### Comparison of methods to estimate fecundity

Assuming that breeding densities of our study populations are high enough to elicit density dependent regulation of fecundity, we expect the model-based estimates of fecundity (i.e., those computed from the component reproductive rates estimated, presumably, at low density) to be higher than fecundity estimates based on adjusted harvest age ratios. Comparison of age ratio-based and model-based fecundity estimates (Fig 2) revealed that model-based fecundity estimates were greater or similar only for eastern black scoter, eastern surf scoter, and western surf scoter. The reverse was true for white-winged scoter, long-tailed duck, and American common eider. If breeding density in these populations is high enough to elicit density dependent regulation, greater age-ratio based fecundity estimates are disconcerting, and suggests that one, or both, estimates of fecundity may be biased. If recent population declines have reduced breeding densities such that density regulation of fecundity is not occurring, inclusion of age ratio based estimates is reasonable and the discrepancy between age ratios and model-based fecundity estimates would indicate that model-based estimates of fecundity under optimal conditions are biased low.

Even if reflective of optimal growth conditions, age ratio estimates may be biased. National harvest surveys used to estimate age ratios may not provide reliable data at regional or population scales [18] and estimates of observed harvest for some of our study populations may be inaccurate. Seasonal and geographic structuring or segregation of population age or sex cohorts, combined with small, and possibly unrepresentative, samples of hunter-submitted wings could cause bias in harvest age ratio estimates for specific populations [18, 65, 66]. If breeding density is sufficiently high to invoke density dependent regulation then age-ratios based on national harvest surveys and banding data are not reflective of conditions for optimal population growth.

While we requested estimates of component reproductive rates used in our model-based estimates of fecundity during the elicitation, clearly this was challenging for any of these populations, especially given the limited availability of data and published information. We suspect that, in many instances, median estimates of component reproductive rates did not adequately account for the effects of density-dependent regulation and were biased low resulting in an underestimation of maximum fecundity and *r*_max_.

Concerns about fecundity estimates derived using each method, and uncertainty about the degree of density-dependent regulation occurring in these populations, led us to incorporate both independent measures into the overall probability distribution for this parameter. Our results are conditional on both estimation methods being retained in the analysis. If one of the 2 methods were used as the sole source for information on fecundity, the relative sensitivity analysis rankings could change. Including both methods ultimately made the assessment more robust to the considerable uncertainty about fecundity levels expected under optimal growth conditions for each population.

### Comparison of allowable and observed harvest

Our PTL (allowable harvest or harvest rate) estimates were highly uncertain, much more so than the estimates of observed harvest that were based on national harvest survey programs in the U.S. and Canada as well as generally less reliable estimates of subsistence harvest. In fact, for every population examined and for both values of *θ*, the 95% credible interval for observed harvest fell entirely within the 95% credible interval for allowable take. This illustrates that, although a better understanding of the components of observed harvest (bias in fall and winter harvest estimates, comprehensive and up-to-date estimates of subsistence harvest, crippling loss) is desirable, a reduction in uncertainty in allowable harvest would likely be more informative to harvest management. For those populations where subsistence harvest estimates far exceeded sport harvest, a reasonable case can be made for better subsistence harvest estimates.

The percentage of simulations for each of the 7 sea duck populations where observed harvest < allowable harvest (Table 2) provides a general framework for assessing the risk of each population to overharvest. Any conclusions are, of course, conditional on the probability distributions used to characterize uncertainty in each demographic parameter, the assumptions and limitations of the deterministic PTL framework (see *Challenges and Assumptions*), and the assumed management objective of MSY. According to the simulation results, we grouped the 7 populations into 3 categories reflecting the risk of overharvest. The eastern black scoter and western surf scoter populations are at lowest risk of overharvest; eastern surf scoter, western black scoter, and white-winged scoter are at moderate risk; and long-tailed duck and American common eider are at the highest risk of overharvest.

For the populations with lowest risk of overharvest, the proportion of the median fall population size harvested was 0.06 for eastern black scoter and 0.02 for western surf scoter. Comparison of estimates of fecundity for these 2 populations show logical consistency, with model-based estimates exceeding those based on harvest age ratio (though perhaps not by as much as might be expected) and provide reasonable confidence in the median estimate of *r*_max._. Growth potential in these populations appears sufficient to sustain contemporary harvest levels.

Of the populations identified as having moderate risk of overharvest, risk is highest for eastern surf scoter, as is the proportion of the median fall population harvested (0.10). Within this group, only the fecundity estimates for eastern surf scoter show logical consistency such that model-based estimates exceed those from harvest age ratios. No comparison of fecundity estimates was possible for western black scoter due to insufficient wing samples. For white-winged scoter, the median fecundity estimate from harvest age ratios exceeds the median model-based estimate suggesting that one, or both, estimates may be biased, again if it is assumed that these populations are undergoing density dependent regulation of fecundity. The difference between the 2 fecundity medians is so great for white-winged scoter that we suspect both the estimates are biased, in opposite directions. With a relatively low proportion of this population estimated to be harvested annually, the risk of overharvest is driven primarily by the low combined estimate of fecundity.

Our assessment suggests that American common eider and long-tailed duck are at the greatest risk of overharvest; median estimated *r*_max_ for both populations is less than, or near, 0, indicative of no long-term growth potential even in the absence of sport and subsistence harvest. In our assessment of allowable harvest of American common eider, most of the information used to describe fecundity was based on field observations and experts from Maine and the Canadian Maritimes where the population is believed to be in decline. Here several biological hypotheses have been advanced, from gull predation on ducklings to climate-related regime shifts in the Gulf of Maine, which could account for a low *r*_max_ and low growth potential. Our assessment indicates that although harvest rates of hen American common eiders were low, the harvest potential of this subspecies, given current environmental conditions and other anthropogenic stressors, might be even lower. This conclusion may apply only to the portion of the population breeding in Maine and the Maritimes, where demographic information was available.

Based on limited breeding population data from surveys that covered only a small fraction of the breeding range of long-tailed duck in North America, Bowman et al. [11] reported evidence that this species declined during the 1980s and 1990s, but has stabilized or increased since the early 2000s. Of the populations examined in this study, monitoring capacity and population estimates are poorest for long-tailed ducks which breed at low density in remote arctic habitats not covered by breeding population surveys. Large-scale winter population estimates have only recently become available and cannot be used to assess population trends. In addition, winter surveys have not covered the entire species winter range. Available published estimates for a limited number of reproductive parameters for this species are also very sparse and may apply only to local study populations. To date, no biological hypothesis has been advanced that would corroborate an ongoing high rate of population decline consistent with our median estimate of *r*_max_.

Finally, we note that our assessment of risk applies to overharvest and population decline. We recognize that there is also socio-economic risk associated with loss of harvest opportunity that must be considered in regulatory decision-making.

### Sensitivity of harvest comparisons and influential parameters

Uncertainty in fecundity had more influence on comparisons of allowable and observed harvest than survival, population size, or observed harvest (or harvest rate). A mechanistic understanding of the fecundity process, as would be supported through study of component reproductive rates, may be useful in directing management resources. For several populations, uncertainty in individual reproductive rates greatly influenced inferences about harvest levels. In particular, duckling survival and nest success were influential to inferences about overharvest risk for most populations. Estimating the true population means for individual reproductive parameters, however, will be challenging for any population though technological advances in remote monitoring and physiological data logging may make large-scale studies more feasible. Replicated local-scale studies of component reproductive processes, distributed across the breeding distribution of individual populations, may also have great value by helping elucidate the relationship between these processes and population density.

Likewise, uncertainty in *DV* was influential to harvest comparisons for most populations. In the short-term, improved estimates of fecundity based on female age ratios, adjusted by improved estimates of *DV*, may be a more tractable solution than estimation of individual recruitment parameters at the population scale. Therefore, we recommend increased efforts to improve estimates of *DV* and adjusted harvest age ratios of female sea ducks. This would require increased preseason banding efforts, or some other novel method, to develop sea duck-specific estimates of *DV*. In some instances, population specific estimates of differential vulnerability may be necessary. For example, for American common eiders, differential vulnerability is suspected to vary between the U.S. and Canada in relation to cultural differences affecting the targeting of different cohorts by sport hunters. In the U.S., eider hunting is perceived more as trophy hunting, focused on adult males, and a large proportion of the harvest occurs through guided hunts. In Canada less trophy-status is afforded this species and higher rates of female and hatch-year male harvest occur [18]. Differences in vulnerability of the female age classes in relation to sport and subsistence harvest should also receive attention. Efforts to increase the sample of sea duck wings obtained during annual harvest surveys in the U.S. and Canada would also be necessary to improve the utility of adjusted harvest age ratios as measures of fecundity for these populations. Increasing the wing sample is especially critical, and would be particularly difficult in the Pacific Flyway, where sea duck harvest is low and sample sizes of hunter-submitted wings are small. In this assessment, small samples sizes of hunter-submitted wings precluded the consideration of the age-ratio based method for western black scoters.

Other methods of estimating population age ratio such as those based on direct observation of age and sex ratio of flocks during fall and winter [66, 67] should also be explored. Spatial and density-related variation in these observed ratios may, however, be related to differential migration and segregation. Broad-scale sampling or a better understanding of factors affecting cohort segregation would be required to ensure representativeness.

Finally, since age ratios are not necessarily estimated under ideal growth conditions, a method of projecting age ratios that would be expected under conditions of maximum population growth would need to be devised. Hierarchical models could be formulated to estimate fecundity at low density as a latent effect, however, such approaches require time series of population estimates or other measures of density that are generally lacking for sea ducks. It is also unclear whether, given sea duck life history strategies that favor population stability, sufficient variation in density would be detected from population surveys to estimate the latent effect. Finally, introduced or colonizing populations might be studied to estimate age ratios at low population density.

Our comparisons of allowable and observed harvest were also highly influenced by adult survival in all populations. This was expected, given the life history characteristics of sea ducks and the structure of population projection models [68]. With the notable exception of western surf scoter, the relative proportional uncertainty of adult survival was small in comparison to other parameters, reflecting expert perceptions that there is low uncertainty about the true population mean values. Despite less perceived uncertainty by the elicitation panel in adult survival for most populations, data to estimate survival are still sparse in comparison to some other waterfowl. The high sensitivity of growth rate to adult female survival argues in favor of increased efforts to estimate this critical parameter and verify that median values and uncertainty distributions used in the assessment are reasonable. We recognize the challenges in banding sufficient preseason samples of adult female sea ducks and that estimation based on other banding periods or based on satellite telemetry data may be more feasible. Efforts to verify and scale published allometric relationships between body size and adult survival [22] for sea ducks may also be of value if estimation of adult female survival proves intractable for some species.

Uncertainty about population size was also influential to harvest inferences in 5 of the 6 populations where it factored into the assessment. Bowman et al. [11] recently reviewed population status and monitoring programs for North American sea ducks. In general, but with some exceptions, current waterfowl breeding population monitoring programs are poorly timed to sea duck breeding chronology, suffer from poorly understood species misclassification biases (especially for scoters), and poorly account for detection and availability biases [11]. Bowman et al. [11] reported that little information exists to assess breeding population status or trend for the eastern black scoter given a breeding range that poorly coincides with the spatial extent of population monitoring programs. Similarly, population monitoring programs are not ideally designed spatially for western surf scoter and limited data suggest some discrepancies among regional trends in breeding and wintering populations. The breeding range of eastern surf scoter corresponds better to the spatial extent of current monitoring programs and indications from limited data are that this population has been stable or increasing since 1990 [11]. Lastly, the vast and remote arctic breeding range occupied by the long-tailed duck has prevented the development of adequate breeding population abundance monitoring programs for this species. Although available resources may preclude major shifts in annual spatial coverage or timing of current survey efforts, it is possible that new procedures to accommodate climate change or address differences in waterfowl breeding chronologies could help address some limitations to breeding sea duck monitoring protocols. We recommend continued efforts to integrate the operating procedures and analysis of presently disparate breeding population surveys for sea ducks.

Winter population surveys are receiving increased attention as alternative means of improving sea duck monitoring capacity. Significant challenges with aggregated and variable distributions, mixed species flocks, species misclassification, and limited information on detection and availability processes hamper interpretation of winter surveys [49, 60] However, given the importance of uncertainty in population size estimates to harvest inferences, we recommend continued efforts to improve winter population survey count data. Remote sensing-based designs offer promise in addressing many challenges of both wintering and breeding surveys, as well as increasing safety of survey personnel.

Uncertainty about observed harvest estimates was influential on harvest inferences for only two populations: western black scoter and white-winged scoter. Nearly all of the western black scoter harvest results from subsistence hunting in Alaska, where harvest survey methodology relies on hunters to identify species correctly when they report their harvest. Rothe et al. [18] stated that some of Alaska’s subsistence hunters probably report all scoters they harvest as black scoters, thereby inflating harvest estimates for that species. Efforts are currently underway to improve the Alaska subsistence harvest survey, which could result in reduced uncertainty around western black scoter harvest estimates. Continental harvest of white-winged scoters is less than that of any other species we assessed; thus, harvest estimates for that species would benefit from larger wing samples, as recommended above.

### Challenges and assumptions

The most difficult aspect of our assessment was specifying distributions for demographic parameters. As we described above, demographic rates used in the estimation of *r*_max_ are rarely observed in nature, particularly in harvested populations. Estimates of *r*_max_ for harvest management applications ideally account for the mean environmental conditions and anthropogenic stressors that set upper limits on growth rate for a particular population [21, 32, 33]. While, through the elicitation, we sought to obtain estimates of demographic rates that reflected maximum growth potential for each population, we acknowledge the difficulty in doing so, and that survival and reproductive parameters and *r*_max_ may be under-estimated.

In contrast, the DIM-based estimates of *r*_max_ are based on allometric relationships and relationships among survival, fecundity, and generation time that apply broadly within taxonomic groups. We view the DIM-based estimates as theoretical maximum values of *r*_max_ for each species which may overestimate harvest potential for a specific population under prevailing environmental conditions and other anthropogenic stressors. Our estimates of population-specific *r*_max_ were lower than the species’ theoretical maximum values of *r*_max_ in all cases (Table 2). For white-winged scoter, American common eider, and long-tailed duck, the differences between population-specific and theoretical maximum values of *r*_max_ were largest. It is not possible to determine if these differences are due to suboptimal environmental conditions or anthropogenic factors, to bias in estimates of demographic rates, or both.

Estimating the functional form of the relationship between population density, growth rate, and K as described by the parameter *θ* in the theta-logistic growth model is also challenging. Theta is most commonly estimated from a time series of population indices which are lacking for sea ducks [29, 69, 70]. Because we thought it would be difficult for experts to provide informed judgments regarding values of *θ* for any of these species/populations, we bracketed *θ* based on a published estimate for a North American waterfowl species with similar life history and ran the simulations for *θ* = 1 and *θ* = 2. Given the life history characteristics of sea ducks, it is likely that true values of *θ* are >1 and that density dependence in these species is strongest as population size approaches K. The value of *θ* can have significant implications for harvest potential [22], increasing allowable take levels as the population approaches K for more K-selected species. If *θ* is > 2 for the populations that we simulated, our assessment will underestimate harvest potential. There are a number of other important assumptions implicit to PTL that challenge its application to harvest management. First, PTL assumes that K is not changing. Carrying capacity, however, varies in response to environmental variation or to directional system changes. PTL is robust to changes in carrying capacity as long as *F*_*0*_ is formulated to maintain the population at a fixed fraction of K. This can be accomplished through a harvest rate formulation of PTL or through a time-specific, total harvest-based formulation which scales allowable harvest in accordance with population size [8, 22]. For the latter, it is critical that periodically updated population estimates are available in order to adjust PTL.

Second, PTL assumes *r*_max_ is fixed. Since we view *r*_max_ as a reflection of species life history traits expressed within a specific environmental/anthropogenic setting, it is possible that *r*_max_ could change over time as mean conditions change. There is no remedy for this problem other than conservatism in decision-making and vigilance in monitoring [8] to ensure that periodic updating of *r*_max_ estimates is possible.

Third, PTL as formulated assumes that hunting mortality is additive to other forms of mortality. This is a conservative assumption; however, PTL can be re-framed to allow for partial compensation [29]. Given the life history characteristics of sea ducks, and in the interest of simplification, we did not consider compensation or partial compensation of harvest mortality.

Finally, PTL, as applied here, is based on a highly simplified model of population growth: the deterministic theta-logistic model. While the methods we used to estimate *r*_max_ allow for limited incorporation of age/stage structure, sparse monitoring data for sea ducks limits rigorous examination of the effects of age/stage structure, population inertia/transient dynamics, environmental and other stochastic effects, cohort-targeted harvest, and other factors affecting the dynamics of structured populations. PTL was selected as an initial assessment framework because the simplicity of the underlying model permits broad application to a variety of sea duck species.

## Conclusions and future development

While this assessment highlights a high degree of uncertainty in allowable harvest, it provides a framework for integration of new data from future research and monitoring. It could also serve as the basis for harvest strategy development as questions about objectives, regulatory packages, and the relationship between regulations and harvest are resolved.

We recommend that these PTL assessments be replicated at some reasonable time interval, incorporating additional information derived through research and monitoring. This could be accomplished in a number of ways including repeating the elicitation, updating or incorporating distributions based on empirical data, and/or weighting distributions. The results and conclusions of this study are conditional on the uncertainty described in the demographic parameters for each of the 7 populations. Changing the uncertainty distribution of one parameter can affect the relative sensitivity of harvest inferences to that parameter, as well as others, and would be expected to change the monitoring and research priorities described here. We note that this assessment framework could also be used in a more prospective manner. By speculating about the expected reduction in uncertainty in individual parameters that is anticipated from specific research and monitoring proposals, the funding agencies or partnerships such as the SDJV could use this framework to contrast individual proposals based on their expected effect on the comparison of allowable and observed harvest.

A more formal analysis of the effect of uncertainty and its reduction on harvest decisions could be achieved through an assessment of the expected value of perfect information (EVPI) or expected value of partial information (EVPXI) [9, 71]. These approaches require a fully-specified decision framework to include a harvest management objective, a set of actions (different regulatory alternatives in a harvest management context), and system and control models that specify the effects of regulatory actions and uncertainty about those effects. While a PTL assessment could provide a component of this larger decision framework, other elements, some technical, some policy-based, are presently unspecified. Efforts are underway to specify these policy aspects, further develop decision frameworks to guide sea duck harvest management, and apply more formal approaches to prioritize research and monitoring.

## Supporting information

S1 File Derivation of harvest rate (h_MSY_) and absolute harvest (H_MSY_). (DOCX)

S2 File Data and expert elicitation results for parameters used in the assessment. (XLSX)

S3 File R-code for simulations. (R)

S1 Table Parameter estimates provided to the expert elicitation panel as background material. (DOCX)

S2 Table Sensitivity of harvest difference to individual demographic parameters. (XLSX)

## References

[1] Artelle KA, Anderson SC, Cooper AB, Paquet PC, Reynolds JD, and Darimont CT. Confronting uncertainty in wildlife management: performance of grizzly bear management. PLoS ONE. 2013; 8(11): e78041 doi: 10.1371/journal.pone.0078041 24223134 10.1371/journal.pone.0078041 PMC3819331

[2] Bunnefeld N, Hoshino E, Milner-Gulland EJ. Management strategy evaluation: a powerful tool for conservation? Trends in Ecology and Evolution. 2011; 26: 441–447 doi: 10.1016/j.tree.2011.05.003 21680051 10.1016/j.tree.2011.05.003

[3] Harwood J, Stokes K. Coping with uncertainty in ecological advice: lessons from fisheries. Trends in Ecology and Evolution. 2003; 18:617–622.

[4] Hilborn R, Walters CJ. Quantitative fisheries stock assessment: choices, dynamics, and uncertainty Routledge, Chapman, and Hall, New York, New York, USA 1992.

[5] Holt CA, Peterman RM. Missing the target: uncertainties in achieving management goals in fisheries on Fraser River, British Columbia, sockeye salmon (*Oncorhychus nerka*). Canadian Journal of Fisheries and Aquatic Science. 2006; 63:2722–2733.

[6] Johnson FA, Moore CT, Kendall WL, Dubovsky JA, Caithamer DF, Kelley JR, et al Uncertainty and the management of mallard harvests. Journal of Wildlife Management. 1997; 61:202–216.

[7] Punt AE, Donovan GP. Developing management procedures that are robust to uncertainty: lessons from the International Whaling Commission. ICES Journal of Marine Science. 2007; 64:603–612.

[8] Runge MC, Sauer JR, Avery ML, Blackwell BF, Koneff MD. Assessing allowable take of migratory birds. The Journal of Wildlife Management. 2009;73(4):556–65.

[9] Runge MC, Converse SJ, Lyons JE. Which uncertainty? Using expert elicitation and expected value of information to design an adaptive program. Biological Conservation. 2011; 144:1214–1223.

[10] Walters CJ. Adaptive management of renewable natural resources. MacMillan, New York, New York, USA 1986.

[11] Bowman TD, Silverman ED, Gilliland SG, and Leirness JB. Status and trends of North American sea ducks: Reinforcing the need for better monitoring In: Savard JP, Derksen DV, Esler D, Eadie JM, editors. Ecology and Conservation of North American Sea Ducks. Boca Raton (FL): CRC Press; 2015 pp. 1–28.

[12] Caithamer DF, Otto M, Padding PI, Sauer JR, and Haas GH. Sea ducks in the Atlantic Flyway: Population status and a review of special hunting seasons. U. S. Fish and Wildlife Service, Washington, DC; 2000.

[13] Goudie RI, Brault S, Conant B, Kondratyev AV, Petersen MR, Vermeer K. The status of sea ducks in the North Pacific rim: toward their conservation and management. In: Transactions of the North American Wildlife and Natural Resources Conference. 1994; 59, pp. 27–49.

[14] Kehoe P, Caithamer D, Myers J, Burrel R, Allen B, Haas G, et al Status of sea ducks in the Atlantic Flyway. Canadian Wildlife Service, Fredericton, New Brunswick, Canada; 1994.

[15] Sea Duck Joint Venture Continental Technical Team. Species status reports. U.S. Fish and Wildlife Service, Anchorage, Alaska, USA and Canadian Wildlife Service, Sackville, New Brunswick, Canada; 2003.

[16] Sea Duck Joint Venture Management Board. Sea Duck Joint Venture Strategic Plan 2008–2012. U. S. Fish and Wildlife Service, Anchorage, Alaska, USA, and Canadian Wildlife Service, Sackville, New Brunswick, Canada; 2008.

[17] Boyd WS, Bowman TD, Savard J-PL, Dickson RD. Conservation of North American sea ducks In: Savard JP, Derksen DV, Esler D, Eadie JM, editors. Ecology and Conservation of North American Sea Ducks. Boca Raton (FL): CRC Press; 2015 pp. 529–559.

[18] Rothe TC, Padding PI, Naves LC, Robertson GJ. Harvest of sea ducks in North America: A contemporary summary In: Savard JP, Derksen DV, Esler D, Eadie JM, editors. Ecology and Conservation of North American Sea Ducks. Boca Raton (FL): CRC Press; 2015 pp. 417–467.

[19] Gilliland SG, Gilchrist HG, Rockwell RF, Robertson GJ, Savard JP, Merkel F, et al Evaluating the sustainability of harvest among northern common eiders *Somateria mollissima borealis* in Greenland and Canada. Wildlife Biology. 2009;15(1):24–36.

[20] Krementz DG, Brown PW, Kehoe FP, Houston CS. Population dynamics of white-winged scoters. Journal of Wildlife Management. 1997; 61:222–227.

[21] Runge MC, Kendall WL, Nichols JD. Exploitation In: Sutherland WJ, Newton I, Green R. Bird ecology and conservation: a handbook of techniques. Oxford University Press; 2004.

[22] Johnson FA, Walters MA, Boomer GS. Allowable levels of take for the trade in Nearctic songbirds. Ecological Applications. 2012;22(4):1114–30. 22827122 10.1890/11-1164.1

[23] Wade PR. Calculating limits to the allowable human‐caused mortality of cetaceans and pinnipeds. Marine Mammal Science. 1998;14(1):1–37.

[24] Dooley J, Osnas E., Zimmerman G. Analyses of emperor goose survey data and harvest potential. Report to U.S. Fish and Wildlife Service, Division of Migratory Bird Management, Region 7 and Alaska Migratory Bird Co-Management Council. Anchorage, Alaska, USA. 2016.

[25] Garrettson P. Estimating harvest potential of wood ducks in eastern North America. U.S. Fish and Wildlife Service, Division of Migratory Bird Management. Laurel, Maryland, USA. 2007.

[26] Seamans M, Rivera-Milan F, Koneff M. Estimation of potential biological removal of great black-backed, herring, laughing, and ring-billed gulls from Bird Conservation Regions 15 and 30. U.S. Fish and Wildlife Service, Division of Migratory Bird Management. Laurel, Maryland, USA. 2007.

[27] U.S. Fish and Wildlife Service. Band-tailed pigeon potential take level assessment. Division of Migratory Bird Management, Washington, D.C. USA. 2015.

[28] U.S. Fish and Wildlife Service. Bald and golden eagles: population demographics and estimation of sustainable take in the United States, 2016 update. Division of Migratory Bird Management, Washington, D.C. USA. 2016.

[29] Williams CK. Accounting for wildlife life‐history strategies when modeling stochastic density‐dependent populations: A review. Journal of Wildlife Management. 2013;77(1):4–11.

[30] Boomer GS, Zimmerman GS, Zimpfer NL, Garrettson PR, Koneff MD, Sanders TA, et al Band reporting probabilities for mallards recovered in the United States and Canada. Journal of Wildlife Management. 2013;77(5):1059–66.

[31] Brownie C, Anderson DR, Burnham KP, Robson DR. Statistical inference from band recovery data–A handbook. 2nd ed. U. S. Fish and Wildlife Service Resource Publication 156, Washington, DC; 1985.

[32] Andrewartha HG, Birch LC. The distribution and abundance of animals. University of Chicago Press; 1954.

[33] Caughley G. Analysis of vertebrate populations. New York: John Wiley and Sons; 1977.

[34] Bordage D, Savard JP. Black Scoter (*Melanitta nigra*) In: Rodewald PG, editor. Birds of North America. Ithaca: Cornell Lab of Ornithology; 2011 Available: https://birdsna.org/Species-Account/bna/species/blksco2

[35] Brown PW, Fredrickson LH. White-winged Scoter (*Melanitta fusca*) In: Rodewald PG, editor. Birds of North America. Ithaca: Cornell Lab of Ornithology; 1997 Available: https://birdsna.org/Species-Account/bna/species/whwsco

[36] Goudie RI, Robertson GJ, Reed A. Common Eider (*Somateria mollissima*) In: Rodewald PG, editor. Birds of North America. Ithaca: Cornell Lab of Ornithology; 2000 Available: Available: https://birdsna.org/Species-Account/bna/species/comeid

[37] Robertson GJ, Savard JP. Long-tailed Duck: *Clangula hyemalis* In: Rodewald PG, editor. Birds of North America. Ithaca: Cornell Lab of Ornithology; 2002 Available: https://birdsna.org/Species-Account/bna/species/lotduc

[38] Anderson EM, Dickson RD, Lok EK, Palm EC, Savard J-PL, Bordage D, et al Surf Scoter (*Melanitta perspicillata*) In: Rodewald PG, editor. Birds of North America. Ithaca: Cornell Lab of Ornithology; 2015 Available: https://birdsna.org/Species-Account/bna/species/sursco

[39] Caswell H. Matrix population models: Construction, analysis, and interpretation, 2^nd^ ed. Sunderland (MA): Sinauer; 2001.

[40] Stubben C, Milligan B. Estimating and analyzing demographic models using the popbio package in R. Journal of Statistical Software. 2007;22(11):1–23.

[41] R Core Team. R: A Language and Environment for Statistical Computing, V3.2.4. Vienna, Austria: R Foundation for Statistical Computing; 2014.

[42] Niel C, Lebreton JD. Using demographic invariants to detect overharvested bird populations from incomplete data. Conservation Biology. 2005;19(3):826–35.

[43] Nelson AL, Martin AC. Gamebird weights. The Journal of Wildlife Management 1953;17:36–42.

[44] Korschgen CE. Breeding stress of female eiders in Maine. Journal of Wildlife Management. 1977:360–73.

[45] Bellrose FC. Ducks, Geese and Swans of North America 3rd ed. Washington DC: Wildlife Management Institute; 1980.

[46] Vermeer K, Bourne N. The white-winged scoter diet in British Columbia waters: resource partitioning with other scoters. Marine birds: their feeding ecology and commercial fisheries relationships. 1984:30.

[47] Leafloor JO, Thompson JE, Ankney CD. Body mass and carcass composition of fall migrant oldsquaws. The Wilson Bulletin. 1996:567–72.

[48] Kellett DK, Alisauskas RT, Mehl KR, Drake KL, Traylor JJ, Lawson SL. Body mass of long-tailed ducks (*Clangula hyemalis*) during incubation. The Auk. 2005;122(1):313–8.

[49] Silverman ED, Leirness JB, Saalfeld DT, Koneff MD, Richkus KD. Atlantic coast wintering sea duck survey, 2008–2011 U. S. Fish and Wildlife Service, Laurel (MD); 2012.

[50] McBride MF, Garnett ST, Szabo JK, Burbidge AH, Butchart SH, Christidis L, et al Structured elicitation of expert judgments for threatened species assessment: a case study on a continental scale using email. Methods in Ecology and Evolution. 2012; 3(5):906–20.

[51] Ayyub BM. Elicitation of expert opinions of uncertainty and risks. CRC, Boca Raton, Florida, USA. 2001.

[52] Martin TG, Burgman MA, Fidler F, Kuhnert PM, Low-Choy S, McBride M, et al Eliciting expert knowledge in conservation science. Conservation Biology. 2012; 26:29–38. doi: 10.1111/j.1523-1739.2011.01806.x 22280323 10.1111/j.1523-1739.2011.01806.x

[53] Speirs‐Bridge A, Fidler F, McBride M, Flander L, Cumming G, Burgman M. Reducing overconfidence in the interval judgments of experts. Risk Analysis. 2010;30(3):512–23. doi: 10.1111/j.1539-6924.2009.01337.x 20030766 10.1111/j.1539-6924.2009.01337.x

[54] Conroy MJ, Peterson JT. Decision making in natural resource management: a structured, adaptive approach Oxford: Wiley Blackwell; 2013.

[55] Delignette-Muller ML, Dutang C. fitdistrplus: An R package for fitting distributions. Journal of Statistical Software. 2015;64(4):1–34

[56] Link WA, Barker RJ. Bayesian inference: with ecological applications. London: Academic Press; 2009.

[57] Padding PI, Gobeil JF, Wentworth C. Estimating waterfowl harvest in North America Waterbirds around the world. The Stationery Office, Edinburgh, United Kingdom 2006:849–52.

[58] <j/>Waltho C, Coulson J. The common eider. T & AD Poyser, London, United Kingdom 2015.

[59] Smith GW. A critical review of the aerial and ground surveys of breeding waterfowl in North America. U.S. Department of the Interior, National Biological Service, Biological Science Report 5. Washington, D.C., USA. 1995.

[60] Silverman ED, Saalfeld DT, Leirness JB, Koneff MD. Wintering sea duck distribution along the Atlantic Coast of the United States. Journal of Fish and Wildlife Management. 2013;4(1):178–98.

[61] Padding PI, Royle JA. Assessment of bias in US waterfowl harvest estimates. Wildlife Research. 2012;39(4):336–42.

[62] Natcher DC, Felt L, Chaulk K, Procter A. Monitoring the Domestic Harvest of Migratory Birds in Nunatsiavut, Labrador. Arctic. 2011:362–6.

[63] Tobias TN, Kay JJ. The bush harvest in Pinehouse, Saskatchewan, Canada. Arctic. 1994:207–21.

[64] Krementz DG, Hines JE, Caithamer DF. Survival and recovery rates of American eiders in eastern North America. The Journal of Wildlife Management. 1996:855–62.

[65] Sæther B-E, Engen S. Pattern of variation in avian population growth rates. Philosophical transactions of the Royal Society of London. 2002; 357:1185–1195. doi: 10.1098/rstb.2002.1119 12396511 10.1098/rstb.2002.1119 PMC1693028

[66] Iverson SA, Smith BD, Cooke F. Age and sex distributions of wintering surf scoters: implications for the use of age ratios as an index of recruitment. The Condor. 2004;106(2):252–62.

[67] Rodway MS, Regehr HM, Boyd WS, Iverson SA. Age and sex ratios of sea ducks wintering in the Strait of Georgia, British Columbia: Implications for monitoring. Marine Ornithology. 2015;43:141–50.

[68] Flint PL. Population dynamics of sea ducks In: Savard JP, Derksen DV, Esler D, Eadie JM, editors. Ecology and Conservation of North American Sea Ducks. Boca Raton (FL): CRC Press; 2015 pp. 63–96.

[69] Sæther BE, Engen S, Matthysen E. Demographic characteristics and population dynamical patterns of solitary birds. Science. 2002;295(5562):2070–3. doi: 10.1126/science.1068766 11896278 10.1126/science.1068766

[70] Clark F, Brook BW, Delean S, Akçakaya HR, Bradshaw CJA. The theta‐logistic is unreliable for modelling most census data. Methods in Ecology and Evolution. 2010;1(3):253–62.

[71] Johnson FA, Hagan G, Palmer WE, Kemmerer M. Uncertainty, robustness, and the value of information in managing a population of northern bobwhites. The Journal of Wildlife Management. 2014;78(3):531–9.

