# Supplementary material for: Evaluation of harvest and information needs for North American sea ducks
Source: PLoS One. 2017 Apr 18;12(4):e0175411. doi: 10.1371/journal.pone.0175411 (PMC5395144; doi:10.1371/journal.pone.0175411)
Supplement: S1 File — (DOCX) [file pone.0175411.s001.docx]

S1. Derivation of harvest rate (h_MSY_) and absolute harvest (H_MSY_) for maximum sustained yield under the theta-logistic model for a *post-growth* population. $N_{t}$ is fall population size, $r_{\max}$ is the intrinsic rate of growth, *K* is carrying capacity, *h* is harvest rate, and *t* is year.

$N_{t+1}=N_{t}\left( 1+r_{max}\left( 1-\left( \frac{N_{t}}{K} \right)^{\theta} \right) \right)\left( 1-h \right)$,

where the fall population is:

${N_{t}^{FF}=N}_{t}\left( 1+r_{max}\left( 1-\left( \frac{N_{t}}{K} \right)^{\theta} \right) \right)$.

At equilibrium,

$N_{e}=N_{e}\left( 1+r_{max}\left( 1-\left( \frac{N_{e}}{K} \right)^{\theta} \right) \right)\left( 1-h \right)$,

such that

$N_{e}=K\left( 1-\frac{h}{r_{max}\left( 1-h \right)} \right)^{\frac{1}{\theta}}$.

Harvest at equilibrium is:

$H_{e}=hN_{e}\left( 1+r_{max}\left( 1-\left( \frac{N_{e}}{K} \right)^{\theta} \right) \right)=hK\left( 1-\frac{h}{r_{max}\left( 1-h \right)} \right)^{\frac{1}{\theta}}\left( 1+r_{max}\left( 1-\left( \frac{K\left( 1-\frac{h}{r_{max}\left( 1-h \right)} \right)^{\frac{1}{\theta}}}{K} \right)^{\theta} \right) \right)$,

which simplifies to:

$H_{e}=\left( \frac{h}{1-h} \right)K\left( 1-\frac{h}{r_{max}\left( 1-h \right)} \right)^{\frac{1}{\theta}}$.

The maximum sustained yield with respect to harvest rate is found by setting the derivative to zero:

$\frac{dH_{e}}{dh}=\frac{K}{\left( 1-h \right)^{2}}\left( 1-\frac{h}{r_{max}\left( 1-h \right)} \right)^{\frac{1}{\theta}}\left( \frac{r_{max}\theta-h\left( r_{max}\theta+\theta+1 \right)}{\theta\left( r_{max}\left( 1-h \right)-h \right)} \right)=0$,

and solving for *h*:

$h_{MSY}^{FF}=\frac{r_{max}\theta}{1+\theta\left( 1+r_{max} \right)}$.

Then, the associated harvest and population size are:

$N_{MSY}=K\left( \frac{1}{1+\theta} \right)^{\frac{1}{\theta}}$, and

$H_{MSY}=\frac{r_{max}\theta}{1+\theta}K\left( \frac{1}{1+\theta} \right)^{\frac{1}{\theta}}$.
