# Supplementary material for: Evaluation of harvest and information needs for North American sea ducks
Source: PLoS One. 2017 Apr 18;12(4):e0175411. doi: 10.1371/journal.pone.0175411 (PMC5395144; doi:10.1371/journal.pone.0175411)
Supplement: S1 Table — (DOCX) [file pone.0175411.s004.docx]

S1 Table. Demographic parameter estimates derived from published literature and unpublished data provided to the expert elicitation panel as background material.

American common eider

| **Parameter** | **Estimate and variability** | **Scale** | **Source** |
| --- | --- | --- | --- |
| Breeding propensity at age 1 | 0 | Eastern North America | Gilliland SG, Gilchrist HG, Rockwell RF, Robertson GJ, Savard JP, Merkel F, Mosbech A. Evaluating the sustainability of harvest among northern common eiders *Somateria mollissima borealis* in Greenland and Canada. Wildlife Biology. 2009;15(1):24-36. |
| Breeding propensity at age 2 | 0.22 | Eastern North America | Gilliland SG, Gilchrist HG, Rockwell RF, Robertson GJ, Savard JP, Merkel F, Mosbech A. Evaluating the sustainability of harvest among northern common eiders *Somateria mollissima borealis* in Greenland and Canada. Wildlife Biology. 2009;15(1):24-36. |
| Breeding propensity at age 3 | 0.40 | Eastern North America | Gilliland SG, Gilchrist HG, Rockwell RF, Robertson GJ, Savard JP, Merkel F, Mosbech A. Evaluating the sustainability of harvest among northern common eiders *Somateria mollissima borealis* in Greenland and Canada. Wildlife Biology. 2009;15(1):24-36. |
| Breeding propensity at age >4 | 1 | Eastern North America | Gilliland SG, Gilchrist HG, Rockwell RF, Robertson GJ, Savard JP, Merkel F, Mosbech A. Evaluating the sustainability of harvest among northern common eiders *Somateria mollissima borealis* in Greenland and Canada. Wildlife Biology. 2009;15(1):24-36. |
| Clutch size | $\bar{x}$ = 4, range 1-8 | Species | Goudie, RI, Robertson GJ, Reed A. Common Eider (Somateria mollissima). In: Rodewald PG, editor. Birds of North America. Ithaca: Cornell Lab of Ornithology; 2000. |
| Nest success | 0.90 | Eastern North America | Gilliland SG, Gilchrist HG, Rockwell RF, Robertson GJ, Savard JP, Merkel F, Mosbech A. Evaluating the sustainability of harvest among northern common eiders *Somateria mollissima borealis* in Greenland and Canada. Wildlife Biology. 2009;15(1):24-36. |
| Hatching success | 0.90, SE = 0.09 | Eastern North America | Gilliland SG, Gilchrist HG, Rockwell RF, Robertson GJ, Savard JP, Merkel F, Mosbech A. Evaluating the sustainability of harvest among northern common eiders *Somateria mollissima borealis* in Greenland and Canada. Wildlife Biology. 2009;15(1):24-36. |
| 2^nd^ year survival rate | 0.87 | American subspecies | Krementz DG, Hines JE, Caithamer DF. Survival and recovery rates of American eiders in eastern North America. The Journal of Wildlife Management. 1996:855-62. |
| 2^nd^ year survival rate | 0.89 | Maine | B. Allen, unpublished data |
| Adult (>3^rd^ year) survival rate | 0.87 | American subspecies | Krementz DG, Hines JE, Caithamer DF. Survival and recovery rates of American eiders in eastern North America. The Journal of Wildlife Management. 1996:855-62. |
| Adult (>3^rd^ year) survival rate | 0.89 | Maine | B. Allen, unpublished data |
| Adult (>3^rd^ year) survival rate | 0.91, SD = 0.05 | Species | Johnson FA, Walters MA, Boomer GS. Allowable levels of take for the trade in Nearctic songbirds. Ecological Applications. 2012;22(4):1114-30. |
| Age at last breeding | 23 | North America | U.S. Bird Banding Laboratory longevity records (https://www.pwrc.usgs.gov/bbl/longevity/longevity_main.cfm) |
| Harvest rate for adult females | 0.027, SE = 0.002  0.018, 95%CI (0.016-0.021) | Maine (molting)  Maine-So. Labrador (molting+nesting) | B. Allen, unpublished data  G. Zimmerman, unpublished data |
|  |  |  |  |

Eastern black scoter

| **Parameter** | **Estimate and variability** | **Scale** | **Source** |
| --- | --- | --- | --- |
| Age at first breeding | 2^nd^ or 3^rd^ year | Species | Bordage D, Savard JP. Black Scoter (*Melanitta nigra*). In: Rodewald PG, editor. Birds of North America. Ithaca: Cornell Lab of Ornithology; 2011. |
| Adult breeding propensity | Unknown, assume 1 | Eastern North America | Bordage D, Savard JP. Black Scoter (*Melanitta nigra*). In: Rodewald PG, editor. Birds of North America. Ithaca: Cornell Lab of Ornithology; 2011. |
| Clutch size | $\bar{x}$= 7.7, SD = 1.4 | Local (Alaska) | Bordage D, Savard JP. Black Scoter (*Melanitta nigra*). In: Rodewald PG, editor. Birds of North America. Ithaca: Cornell Lab of Ornithology; 2011. |
| Clutch size | $\bar{x}$= 8.7, SD = 1.37 | Local (Iceland) | Bengtson SA. Variations in clutch-size in ducks in relation to the food supply. Ibis. 1971;113(4):523-6. |
| Nest Success | 0.83 | Local (Iceland) | Bengtson SA. Variations in clutch-size in ducks in relation to the food supply. Ibis. 1971;113(4):523-6. |
| Hatching success | $\bar{x}$= 0.95 | Local (Iceland) | Bengtson SA. Reproduction and fluctuations in the size of duck populations at Lake Mývatn, Iceland. Oikos. 1972:35-58. |
| Duckling survival to fledging | $\bar{x}$= 0.48 | Local (Iceland) | Bengtson SA. Reproduction and fluctuations in the size of duck populations at Lake Mývatn, Iceland. Oikos. 1972:35-58. |
| Adult survival rate | 0.85, SE = 0.08 | Eastern North America | Gilliland S, McAloney K, Boyd S, Savard J-P, Reed E, Saalfeld D, Perry M. Satellite tracking of Atlantic black scoters: What we have learned in just a few short years. Unpublished Data, Ecology and Conservation of North American Waterfowl Symposium, Memphis (TN); 2013. |
| Adult survival rate | 0.89, SD = 0.05 | Species | Johnson FA, Walters MA, Boomer GS. Allowable levels of take for the trade in Nearctic songbirds. Ecological Applications. 2012;22(4):1114-30. |
| Age at last breeding | 16 | Species | EURING longevity records (http://www.euring.org/data-and-codes/longevity-list) |
| Winter population count | $\bar{x}$= 211,300, CV = 11% | Atlantic coast and Great Lakes | Silverman ED, Leirness JB, Saalfeld DT, Koneff MD, Richkus KD. Atlantic coast wintering sea duck survey, 2008-2011. U. S. Fish and Wildlife Service, Laurel (MD); 2012. |
| Winter population detection rate | 0.416, SD = 0.011 | Winter aerial surveys | Evenson J, Tschaekofske H, Murphie B, Cyra T, Kraege D. Status and summary of the 2013 WDFW winter sea duck aerial survey detectability project – Phase 3. Washington Department of Fish and Wildlife unpublished report; 2013; J. Leirness, unpublished data |
| Retrieved fall and winter harvest, U.S. | $\bar{x} =$12,678, CV = 16% | Atlantic and Mississippi Flyways | USFWS annual harvest reports |
| Retrieved fall and winter harvest, Canada | $\bar{x}$= 2,408, CV = 32% | Atlantic and Mississippi Flyways | Gendron MH, Smith AC. National Harvest Survey. Bird Populations Monitoring, 2014. National Wildlife Research Centre, Canadian Wildlife Service, Ottawa (ON). |
| Retrieved subsistence harvest | $\bar{x}$= 6,280, CV = 70% | Eastern Canada | Natcher DC, Felt L, Chaulk K, Procter A. Monitoring the Domestic Harvest of Migratory Birds in Nunatsiavut, Labrador. Arctic. 2011:362-6; Cree Trappers Association, Cree Regional Authority and Canadian Wildlife Service. Quebec Cree Subsistence Harvest Survey 2005-2008. 2009. Unpublished report, Montreal, Quebec, Canada, 16pp.; C. Lepage, CWS, personal communication; Rothe TC, Padding PI, Naves LC, Robertson GJ. Harvest of sea ducks in North America: A contemporary summary. In: Savard JP, Derksen DV, Esler D, Eadie JM, editors. Ecology and Conservation of North American Sea Ducks. Boca Raton (FL): CRC Press; 2015. pp. 417-467. |
| Crippling loss | 0.3, CV = 7% | Species, North America | Rothe TC, Padding PI, Naves LC, Robertson GJ. Harvest of sea ducks in North America: A contemporary summary. In: Savard JP, Derksen DV, Esler D, Eadie JM, editors. Ecology and Conservation of North American Sea Ducks. Boca Raton (FL): CRC Press; 2015. pp. 417-467. |

Western black scoter

| **Parameter** | **Estimate and variability** | **Scale** | **Source** |
| --- | --- | --- | --- |
| Age at first breeding | 2^nd^ or 3^rd^ year | Species | Bordage D, Savard JP. Black Scoter (*Melanitta nigra*). In: Rodewald PG, editor. Birds of North America. Ithaca: Cornell Lab of Ornithology; 2011. |
| Adult breeding propensity | Unknown, assume 1 | Western North America | Bordage D, Savard JP. Black Scoter (*Melanitta nigra*). In: Rodewald PG, editor. Birds of North America. Ithaca: Cornell Lab of Ornithology; 2011. |
| Clutch size | $\bar{x}$= 8.7, SD = 1.37 | Local (Iceland) | Bengtson SA. Variations in clutch-size in ducks in relation to the food supply. Ibis. 1971;113(4):523-6. |
| Clutch size | $\bar{x}$ = 7.5, 95% CL 7.2-7.6 | Local (Alaska) | Schamber JL, Broerman FJ, Flint PL. Reproductive ecology and habitat use of Pacific black scoters (*Melanitta nigra americana*) nesting on the Yukon-Kuskokwim Delta, Alaska. Waterbirds. 2010;33(2):129-39. |
| Nest success | $\bar{x}$ = 0.83 | Local (Iceland) | Bengtson SA. Reproduction and fluctuations in the size of duck populations at Lake Mývatn, Iceland. Oikos. 1972:35-58. |
| Nest success | 0.02-0.37 | Local (Alaska) | Schamber JL, Broerman FJ, Flint PL. Reproductive ecology and habitat use of Pacific black scoters (*Melanitta nigra americana*) nesting on the Yukon-Kuskokwim Delta, Alaska. Waterbirds. 2010;33(2):129-39. |
| Hatching success | $\bar{x}$= 0.95 | Local (Iceland) | Bengtson SA. Reproduction and fluctuations in the size of duck populations at Lake Mývatn, Iceland. Oikos. 1972:35-58. |
| Duckling survival rate (to fledging) | $\bar{x}$= 0.48 | Local (Iceland) | Bengtson SA. Reproduction and fluctuations in the size of duck populations at Lake Mývatn, Iceland. Oikos. 1972:35-58. |
| Duckling survival rate (to 30 days) | 0.09-0.35 | Local (Alaska) | Schamber JL, Broerman FJ, Flint PL. Reproductive ecology and habitat use of Pacific black scoters (*Melanitta nigra americana*) nesting on the Yukon-Kuskokwim Delta, Alaska. Waterbirds. 2010;33(2):129-39. |
| Adult survival rate | 0.78, 95% CL 0.72-0.84 | Local (Iceland) | Fox AD, Petersen Æ, Frederiksen M. Annual survival and site‐fidelity of breeding female common scoter *Melanitta nigra* at Mývatn, Iceland, 1925–58. Ibis 2003;145(2):E94-E96. |
| Adult survival rate | 0.89, SD = 0.05 | Species | Johnson FA, Walters MA, Boomer GS. Allowable levels of take for the trade in Nearctic songbirds. Ecological Applications. 2012;22(4):1114-30. |
| Age at last breeding | 16 | Species | EURING longevity records (http://www.euring.org/data-and-codes/longevity-list) |
| Winter population count | $\bar{x}$= 100,216 | Pacific coast | D. Kraege, unpublished data |
| Winter population detection rate | 0.416, SD = 0.011 | Winter aerial surveys | Evenson J, Tschaekofske H, Murphie B, Cyra T, Kraege D. Status and summary of the 2013 WDFW winter sea duck aerial survey detectability project – Phase 3. Washington Department of Fish and Wildlife unpublished report; 2013; J. Leirness, unpublished data |
| Retrieved fall and winter harvest | $\bar{x}$= 550, CV = 47% | Pacific Flyway | USFWS annual harvest reports; Gendron and Smith 2014 |
| Retrieved subsistence harvest | $\bar{x}$= 9,606, CV = 70% | Alaska | Rothe TC, Padding PI, Naves LC, Robertson GJ. Harvest of sea ducks in North America: A contemporary summary. In: Savard JP, Derksen DV, Esler D, Eadie JM, editors. Ecology and Conservation of North American Sea Ducks. Boca Raton (FL): CRC Press; 2015. pp. 417-467. |
| Crippling loss | 0.3, CV = 7% | Species, North America | Rothe TC, Padding PI, Naves LC, Robertson GJ. Harvest of sea ducks in North America: A contemporary summary. In: Savard JP, Derksen DV, Esler D, Eadie JM, editors. Ecology and Conservation of North American Sea Ducks. Boca Raton (FL): CRC Press; 2015. pp. 417-467. |

Eastern surf scoter

| **Parameter** | **Estimate and variability** | **Scale** | **Source** |
| --- | --- | --- | --- |
| Age at first breeding | 2^nd^ or 3^rd^ year | Species | Anderson EM, Dickson RD, Lok EK, Palm EC, Savard J-PL, Bordage D, Reed A. Surf Scoter (*Melanitta perspicillata*). In: Rodewald PG, editor. Birds of North America. Ithaca: Cornell Lab of Ornithology; 2015. |
| Adult breeding propensity | Unknown, assume <1 | Eastern North America | Anderson EM, Dickson RD, Lok EK, Palm EC, Savard J-PL, Bordage D, Reed A. Surf Scoter (*Melanitta perspicillata*). In: Rodewald PG, editor. Birds of North America. Ithaca: Cornell Lab of Ornithology; 2015. |
| Clutch size | $\bar{x}$= 7.6, SD = 0.25 | Local (Quebec) | Morrier AL, Lesage L, Reed E, and Savard J-P. Etude sur l’ecologie de la Macreuse a front blanc au lac Malbaie, Reserve des Laurentides—1994–1995. 2008; Technical Report Series No. 301, Canadian Wildlife Service, Quebec Region, Quebec. |
| Nest success | $\bar{x}$ = 0.55 | Local (Quebec) | Morrier AL, Lesage L, Reed E, and Savard J-P. Etude sur l’ecologie de la Macreuse a front blanc au lac Malbaie, Reserve des Laurentides—1994–1995. 1997; Technical Report Series No. 301, Canadian Wildlife Service, Quebec Region, Quebec. |
| Hatching success | $\bar{x}$= 0.97 | Local (Quebec) | Morrier AL, Lesage L, Reed E, and Savard J-P. Etude sur l’ecologie de la Macreuse a front blanc au lac Malbaie, Reserve des Laurentides—1994–1995. 1997; Technical Report Series No. 301, Canadian Wildlife Service, Quebec Region, Quebec |
| Duckling survival to fledging | 0.424, SE = 0.024 | Local (Quebec) | Morrier AL, Lesage L, Reed E, and Savard J-P. Etude sur l’ecologie de la Macreuse a front blanc au lac Malbaie, Reserve des Laurentides—1994–1995. 1997; Technical Report Series No. 301, Canadian Wildlife Service, Quebec Region, Quebec; Lesage L, Reed A, and Savard JPL. Duckling survival and use of space by surf scoter. Bioone 2008. 15:81-88. |
| Adult survival rate | 0.91, SE = 0.053 | Eastern North America | E. Reed, updated analysis of data from Gilliland SG, Reed E, Lepage C, Lesage L, Savard J-PL, McAloney K. Harvest and survival of eastern North America surf scoters. Abstract of presentation at the 4^rd^ North American Sea Duck Conference, Seward (AK); 2011. |
| Adult survival rate | 0.89, SD = 0.05 | Species | Johnson FA, Walters MA, Boomer GS. Allowable levels of take for the trade in Nearctic songbirds. Ecological Applications. 2012;22(4):1114-30. |
| Age at last breeding | 20 | Species | Longevity of similar spp. (BLSC, WWSC) EURING Longevity records (http://www.euring.org/data-and-codes/longevity-list) |
| Winter population count | $\bar{x}$= 150,826, CV = 11% | Atlantic coast and Great Lakes | Silverman ED, Leirness JB, Saalfeld DT, Koneff MD, Richkus KD. Atlantic coast wintering sea duck survey, 2008-2011. U. S. Fish and Wildlife Service, Laurel (MD); 2012. |
| Winter population detection rate | 0.416, SD = 0.011 | Winter aerial surveys | Evenson J, Tschaekofske H, Murphie B, Cyra T, Kraege D. Status and summary of the 2013 WDFW winter sea duck aerial survey detectability project – Phase 3. Washington Department of Fish and Wildlife unpublished report; 2013; J. Leirness, unpublished data |
| Retrieved fall and winter harvest, U.S. | $\bar{x}$= 25,404, CV = 10% | Atlantic and Mississippi Flyways | USFWS annual harvest reports |
| Retrieved fall and winter harvest, Canada | $\bar{x}$= 3,114, CV = 29% | Atlantic and Mississippi Flyways | Gendron MH, Smith AC. National Harvest Survey. Bird Populations Monitoring, 2014. National Wildlife Research Centre, Canadian Wildlife Service, Ottawa (ON). |
| Retrieved subsistence harvest | $\bar{x}$= 3,562, CV = 70% | Eastern Canada | Natcher DC, Felt L, Chaulk K, Procter A. Monitoring the Domestic Harvest of Migratory Birds in Nunatsiavut, Labrador. Arctic. 2011:362-6; Cree Trappers Association, Cree Regional Authority and Canadian Wildlife Service. Quebec Cree Subsistence Harvest Survey 2005-2008. 2009. Unpublished report, Montreal, Quebec, Canada, 16pp.; C. Lepage, CWS, personal communication; Rothe TC, Padding PI, Naves LC, Robertson GJ. Harvest of sea ducks in North America: A contemporary summary. In: Savard JP, Derksen DV, Esler D, Eadie JM, editors. Ecology and Conservation of North American Sea Ducks. Boca Raton (FL): CRC Press; 2015. pp. 417-467. |
| Crippling loss | 0.3, CV = 7% | Species, North America | Rothe TC, Padding PI, Naves LC, Robertson GJ. Harvest of sea ducks in North America: A contemporary summary. In: Savard JP, Derksen DV, Esler D, Eadie JM, editors. Ecology and Conservation of North American Sea Ducks. Boca Raton (FL): CRC Press; 2015. pp. 417-467. |

Western surf scoter

| **Parameter** | **Estimate and variability** | **Scale** | **Source** |
| --- | --- | --- | --- |
| Age at first breeding | 2^nd^ or 3^rd^ year | Species | Anderson EM, Dickson RD, Lok EK, Palm EC, Savard J-PL, Bordage D, Reed A. Surf Scoter (*Melanitta perspicillata*). In: Rodewald PG, editor. Birds of North America. Ithaca: Cornell Lab of Ornithology; 2015. |
| Adult breeding propensity | Unknown, assume <1 | Eastern North America | Anderson EM, Dickson RD, Lok EK, Palm EC, Savard J-PL, Bordage D, Reed A. Surf Scoter (*Melanitta perspicillata*). In: Rodewald PG, editor. Birds of North America. Ithaca: Cornell Lab of Ornithology; 2015. |
| Clutch size | $\bar{x}$ = 7.6, SD = 0.25 | Local (Quebec) | Morrier AL, Lesage L, Reed E, and Savard J-P. Etude sur l’ecologie de la Macreuse a front blanc au lac Malbaie, Reserve des Laurentides—1994–1995. 1997; Technical Report Series No. 301, Canadian Wildlife Service, Quebec Region, Quebec. |
| Nest success | $\bar{x}$ = 0.55 | Local (Quebec) | Morrier AL, Lesage L, Reed E, and Savard J-P. Etude sur l’ecologie de la Macreuse a front blanc au lac Malbaie, Reserve des Laurentides—1994–1995. 1997; Technical Report Series No. 301, Canadian Wildlife Service, Quebec Region, Quebec. |
| Hatching success | $\bar{x}$= 0.97 | Local (Quebec) | Morrier AL, Lesage L, Reed E, and Savard J-P. Etude sur l’ecologie de la Macreuse a front blanc au lac Malbaie, Reserve des Laurentides—1994–1995. 1997; Technical Report Series No. 301, Canadian Wildlife Service, Quebec Region, Quebec. |
| Duckling survival to fledging | 0.424, SE = 0.024 | Local (Quebec) | Morrier AL, Lesage L, Reed E, and Savard J-P. Etude sur l’ecologie de la Macreuse a front blanc au lac Malbaie, Reserve des Laurentides—1994–1995. 1997; Technical Report Series No. 301, Canadian Wildlife Service, Quebec Region, Quebec; Lesage L, Reed A, and Savard JPL. Duckling survival and use of space by surf scoter. Bioone 2008. 15:81-88. |
| Adult survival rate | 0.91, SE = 0.073 | Eastern North America | D. Kraege, unpublished data |
| Adult survival rate | 0.89, SD = 0.05 | Species | Johnson FA, Walters MA, Boomer GS. Allowable levels of take for the trade in Nearctic songbirds. Ecological Applications. 2012;22(4):1114-30. |
| Age at last breeding | 20 | Species | Longevity of similar spp. (BLSC, WWSC) EURING Longevity records (http://www.euring.org/data-and-codes/longevity-list) |
| Winter population count | $\bar{x}$= 222,983 | Pacific coast | D. Kraege, unpublished data |
| Winter population detection rate | 0.416, SD = 0.011 | Winter aerial surveys | Evenson J, Tschaekofske H, Murphie B, Cyra T, Kraege D. Status and summary of the 2013 WDFW winter sea duck aerial survey detectability project – Phase 3. Washington Department of Fish and Wildlife unpublished report; 2013; J. Leirness, unpublished data |
| Retrieved fall and winter harvest | $\bar{x}$= 4,798, CV = 37% | Pacific Flyway | USFWS annual harvest reports; Gendron MH, Smith AC. National Harvest Survey. Bird Populations Monitoring, 2014. National Wildlife Research Centre, Canadian Wildlife Service, Ottawa (ON). |
| Retrieved subsistence harvest | $\bar{x}$= 2,809, CV = 70% | Alaska | Rothe TC, Padding PI, Naves LC, Robertson GJ. Harvest of sea ducks in North America: A contemporary summary. In: Savard JP, Derksen DV, Esler D, Eadie JM, editors. Ecology and Conservation of North American Sea Ducks. Boca Raton (FL): CRC Press; 2015. pp. 417-467. |
| Crippling loss | 0.3, CV = 7% | Species, North America | Rothe TC, Padding PI, Naves LC, Robertson GJ. Harvest of sea ducks in North America: A contemporary summary. In: Savard JP, Derksen DV, Esler D, Eadie JM, editors. Ecology and Conservation of North American Sea Ducks. Boca Raton (FL): CRC Press; 2015. pp. 417-467. |

White-winged scoter

| **Parameter** | **Estimate and variability** | **Scale** | **Source** |
| --- | --- | --- | --- |
| Age at first breeding | 2^nd^ or 3^rd^ year | Species | Brown PW, Fredrickson LH. White-winged Scoter (Melanitta fusca). In: Rodewald PG, editor. Birds of North America. Ithaca: Cornell Lab of Ornithology; 1997. |
| Adult breeding propensity | 0.72 | Local (Alaska) | Safine DE. Breeding ecology of white-winged scoters on the Yukon Flats, Alaska [thesis]. Fairbanks (AK): University of Alaska; 2005. |
| Clutch size ^1^ | $\bar{x}$ = 8.8, 95% CL 8.6-9.1 | Local (Saskatchewan) | Traylor JJ, Alisauskas RT, Kehoe FP. Nesting ecology of white-winged scoters (*Melanitta fusca deglandi*) at Redberry Lake, Saskatchewan. The Auk. 2004;121(3):950-62. |
| Clutch size ^1^ | $\bar{x}$= 8.1, SE = 0.2 | Local (Alaska) | Safine DE. Breeding ecology of white-winged scoters on the Yukon Flats, Alaska [thesis]. Fairbanks (AK): University of Alaska; 2005. |
| Nest success ^1^ | $\bar{x}$ = 0.24, 95% CI 0.09-0.42 | Local (Saskatchewan) | Traylor JJ, Alisauskas RT, Kehoe FP. Nesting ecology of white-winged scoters (*Melanitta fusca deglandi*) at Redberry Lake, Saskatchewan. The Auk. 2004;121(3):950-62. |
| Nest success ^1^ | $\bar{x}$ = 0.25, 95% CL 0.13-0.49 | Local (Alaska) | Safine DE. Breeding ecology of white-winged scoters on the Yukon Flats, Alaska [thesis]. Fairbanks (AK): University of Alaska; 2005. |
| Hatching success ^1^ | $\bar{x}$= 0.85, 95% CL 0.81-0.88 | Local (Saskatchewan) | Traylor JJ, Alisauskas RT, Kehoe FP. Nesting ecology of white-winged scoters (*Melanitta fusca deglandi*) at Redberry Lake, Saskatchewan. The Auk. 2004;121(3):950-62. |
| Hatching success ^1^ | $\bar{x}$= 0.92, SE = 0.02 | Local (Alaska) | Safine DE. Breeding ecology of white-winged scoters on the Yukon Flats, Alaska [thesis]. Fairbanks (AK): University of Alaska; 2005. |
| Duckling survival rate (to fledging) | 0.18-0.40, $\bar{x}$= 0.28 | Local (Saskatchewan, 1977-80) | Brown PW, Frederickson LH. White-winged Scoter, *Melanitta fusca*, populations and nesting on Redberry Lake, Saskatchewan. Canadian Field-Naturalist. Ottawa (ON). 1989;103(2):240-7. |
| Duckling survival rate (to 30 days) | $\bar{x}$ = 0.0044 | Local (Saskatchewan, 2000-01) | Traylor JJ, Alisauskas RT. Effects of intrinsic and extrinsic factors on survival of white-winged scoter (*Melanitta fusca deglandi*) ducklings. The Auk. 2006;123(1):67-81. |
| Duckling survival rate (to 30 days) | 0.08, 95% CL 0.03-0.15 | Local (Alaska, 2003) | Safine DE. Breeding ecology of white-winged scoters on the Yukon Flats, Alaska [thesis]. Fairbanks (AK): University of Alaska; 2005. |
| Duckling survival rate (to 30 days) | 0.64, 95% CL 0.51-0.75 | Local (Alaska, 2004) | Safine DE. Breeding ecology of white-winged scoters on the Yukon Flats, Alaska [thesis]. Fairbanks (AK): University of Alaska; 2005. |
| Adult survival rate ^1^ | 0.69, SE = 0.04 | Local (Northwest Territories) | Slattery S, Clark B. Apparent annual survival of lesser scaup and white-winged scoter females from the Canadian western boreal forest. 2008; Abstract of presentation at the 3^rd^ North American Sea Duck Conference, Quebec City, Quebec. |
| Adult survival rate ^1^ | 0.84, 95% CL 0.77-0.91 | Local (Saskatchewan) | Alisauskas RT, Traylor JJ, Swoboda CJ, Kehoe FP. Components of population growth rate for White–winged Scoters in Saskatchewan, Canada. Animal Biodiversity and Conservation. 2004;27(1):451-60. |
| Adult survival rate | 0.90, SD = 0.05 | Species | Johnson FA, Walters MA, Boomer GS. Allowable levels of take for the trade in Nearctic songbirds. Ecological Applications. 2012;22(4):1114-30. |
| Age at last breeding | 21 | Species | EURING longevity records |
| Winter population count | $\bar{x}$= 161,890 (Pacific coast) + 58,600, CV = 19% (Atlantic coast) | North America | Silverman ED, Leirness JB, Saalfeld DT, Koneff MD, Richkus KD. Atlantic coast wintering sea duck survey, 2008-2011. U. S. Fish and Wildlife Service, Laurel (MD); 2012; D. Kraege, unpublished data |
| Winter population detection rate | 0.416, SD = 0.011 | Winter aerial surveys | Evenson J, Tschaekofske H, Murphie B, Cyra T, Kraege D. Status and summary of the 2013 WDFW winter sea duck aerial survey detectability project – Phase 3. Washington Department of Fish and Wildlife unpublished report; 2013; J. Leirness, unpublished data |
| Retrieved fall and winter harvest, U.S. | $\bar{x}$= 8,379, CV = 22% | United States | USFWS annual harvest reports |
| Retrieved fall and winter harvest, Canada | $\bar{x}$= 1,891, CV = 32% | Canada | Gendron MH, Smith AC. National Harvest Survey. Bird Populations Monitoring, 2014. National Wildlife Research Centre, Canadian Wildlife Service, Ottawa (ON). |
| Retrieved subsistence harvest | $\bar{x}$= 1,871, CV = 70% | North America | Natcher DC, Felt L, Chaulk K, Procter A. Monitoring the Domestic Harvest of Migratory Birds in Nunatsiavut, Labrador. Arctic. 2011:362-6; Cree Trappers Association, Cree Regional Authority and Canadian Wildlife Service. Quebec Cree Subsistence Harvest Survey 2005-2008. 2009. Unpublished report, Montreal, Quebec, Canada, 16pp.; C. Lepage, CWS, personal communication; Rothe TC, Padding PI, Naves LC, Robertson GJ. Harvest of sea ducks in North America: A contemporary summary. In: Savard JP, Derksen DV, Esler D, Eadie JM, editors. Ecology and Conservation of North American Sea Ducks. Boca Raton (FL): CRC Press; 2015. pp. 417-467. |
| Crippling loss | 0.3, CV = 7% | Species, North America | Rothe TC, Padding PI, Naves LC, Robertson GJ. Harvest of sea ducks in North America: A contemporary summary. In: Savard JP, Derksen DV, Esler D, Eadie JM, editors. Ecology and Conservation of North American Sea Ducks. Boca Raton (FL): CRC Press; 2015. pp. 417-467. |

^1^ Additional estimates from older studies are given and referenced in Brown PW, Frederickson LH. White-winged scoter. No. 274 In: Poole A, Gill F., editors, The Birds of North America. Cornell Laboratory of Ornithology and The Academy of Natural Sciences of Philadelphia. 1997. 25pp.

Long-tailed duck

| **Parameter** | **Estimate and variability** | **Scale** | **Source** |
| --- | --- | --- | --- |
| Age at first breeding | 2^nd^ year | Species | Robertson GJ, Savard JP. Long-tailed Duck: *Clangula hyemalis*. In: Rodewald PG, editor. Birds of North America. Ithaca: Cornell Lab of Ornithology; 2002. |
| Adult breeding propensity | Unknown, assume <1 | North America | Robertson GJ, Savard JP. Long-tailed Duck: *Clangula hyemalis*. In: Rodewald PG, editor. Birds of North America. Ithaca: Cornell Lab of Ornithology; 2002. |
| Clutch size^2^ | $\bar{x}$= 7.1, 95% CL 6.5-7.7 | Local (Alaska) | Schamber JL, Flint PL, Grand JB, Wilson HM, Morse JA. Population dynamics of long-tailed ducks breeding on the Yukon-Kuskokwim Delta, Alaska. Arctic. 2009:190-200. |
| Nest success^2^ | $\bar{x}$ = 0.30, 95% CL 0.24-0.36 | Local (Alaska) | Schamber JL, Flint PL, Grand JB, Wilson HM, Morse JA. Population dynamics of long-tailed ducks breeding on the Yukon-Kuskokwim Delta, Alaska. Arctic. 2009:190-200. |
| Nest success^2^ | 0.14-0.90,$\bar{x}$ = 0.30 | Local (Nunavut) | Kellett DK, Alisauskas RT. Population ecology of long-tailed ducks at Karrak Lake, Nunavut. Unpublished Data, 2014; Fifth International Sea Duck Conference, Rejkavik, Iceland. |
| Nest success^2^ | $\bar{x}$ = 0.589, SE = 0.0505 | Local (Manitoba) | Alison RM. Breeding biology and behavior of the Oldsquaw (*Clangula hyemalis* L.). Ornithological Monographs. 1975(18):i-52. |
| Hatching success | $\bar{x}$= 0.804 | Local (Manitoba) | Alison RM. Breeding biology and behavior of the Oldsquaw (*Clangula hyemalis* L.). Ornithological Monographs. 1975(18):i-52. |
| Duckling survival rate (to 30 days) | 0-0.25, $\bar{x}$ = 0.10 | Local (Alaska) | Schamber JL, Flint PL, Grand JB, Wilson HM, Morse JA. Population dynamics of long-tailed ducks breeding on the Yukon-Kuskokwim Delta, Alaska. Arctic. 2009:190-200. |
| Adult survival rate | 0.74, 95% CL 0.57-0.86 | Local (Alaska) | Schamber JL, Flint PL, Grand JB, Wilson HM, Morse JA. Population dynamics of long-tailed ducks breeding on the Yukon-Kuskokwim Delta, Alaska. Arctic. 2009:190-200. |
| Adult survival rate | 0.85, 95% CL 0.76-0.92 | Local (Nunavut) | Kellett DK, Alisauskas RT, Mehl KR, Drake KL, Traylor JJ, Lawson SL. Body mass of long-tailed ducks (*Clangula hyemalis*) during incubation. The Auk. 2005;122(1):313-8. |
| Adult survival rate | 0.88, SD = 0.05 | Species | Johnson FA, Walters MA, Boomer GS. Allowable levels of take for the trade in Nearctic songbirds. Ecological Applications. 2012;22(4):1114-30. |
| Age at last breeding | 17 | North America | U.S. Bird Banding Laboratory longevity records (https://www.pwrc.usgs.gov/bbl/longevity/longevity_main.cfm) |
| Winter population count | $\bar{x}$= 174,029 (Pacific coast) + 236,552, CV = 11% (Atlantic coast); 53,770 (Great Lakes) | North America | Silverman ED, Leirness JB, Saalfeld DT, Koneff MD, Richkus KD. Atlantic coast wintering sea duck survey, 2008-2011. U. S. Fish and Wildlife Service, Laurel (MD); 2012; D. Kraege, unpublished data; M. Schummer, unpublished data |
| Winter population detection rate | 0.814, SD = 0.034 | Winter aerial surveys | J. Leirness, unpublished data |
| Retrieved fall and winter harvest, U.S. | $\bar{x}$= 26,383, CV = 10% | United States | USFWS annual harvest reports |
| Retrieved fall and winter harvest, Canada | $\bar{x}$= 2,189, CV = 32% | Canada | Gendron MH, Smith AC. National Harvest Survey. Bird Populations Monitoring, 2014. National Wildlife Research Centre, Canadian Wildlife Service, Ottawa (ON). |
| Retrieved subsistence harvest | $\bar{x}$= 9,841, CV = 70% | North America | Natcher DC, Felt L, Chaulk K, Procter A. Monitoring the Domestic Harvest of Migratory Birds in Nunatsiavut, Labrador. Arctic. 2011:362-6; Cree Trappers Association, Cree Regional Authority and Canadian Wildlife Service. Quebec Cree Subsistence Harvest Survey 2005-2008. 2009. Unpublished report, Montreal, Quebec, Canada, 16pp.; C. Lepage, CWS, personal communication; Rothe TC, Padding PI, Naves LC, Robertson GJ. Harvest of sea ducks in North America: A contemporary summary. In: Savard JP, Derksen DV, Esler D, Eadie JM, editors. Ecology and Conservation of North American Sea Ducks. Boca Raton (FL): CRC Press; 2015. pp. 417-467. |
| Crippling loss | 0.3, CV = 7% | Species, North America | Rothe TC, Padding PI, Naves LC, Robertson GJ. Harvest of sea ducks in North America: A contemporary summary. In: Savard JP, Derksen DV, Esler D, Eadie JM, editors. Ecology and Conservation of North American Sea Ducks. Boca Raton (FL): CRC Press; 2015. pp. 417-467. |

^2^ Additional estimates from older studies are given and referenced in Robertson GJ and Savard JPL. Long-tailed duck. No. 651 In: Poole A, Gill F., editors, The Birds of North America. Cornell Laboratory of Ornithology and The Academy of Natural Sciences of Philadelphia. 2002. 27pp.
